# Exploring Genetic Heterogeneity in Type 2 Diabetes Subtypes

**DOI:** 10.3390/genes16101131

**Published:** 2025-09-25

**Authors:** Yanina Timasheva, Olga Kochetova, Zhanna Balkhiyarova, Diana Avzaletdinova, Gulnaz Korytina, Tatiana Kochetova, Arie Nouwen

**Affiliations:** 1 Institute of Biochemistry and Genetics of Ufa Federal Research Centre of Russian Academy of Sciences, 450054 Ufa, Russia; yartimasheva@bashgmu.ru (Y.T.); ovkochetova@bashgmu.ru (O.K.); gfkorytina@bashgmu.ru (G.K.); 2 Department of Medical Genetics and Fundamental Medicine, Faculty of General Medicine, Bashkir State Medical University, 450008 Ufa, Russia; 3 Department of Biology, Faculty of Stomatology, Bashkir State Medical University, 450008 Ufa, Russia; 027620666357@bashgmu.ru; 4 Section of Statistical Multi-Omics, Department of Clinical & Experimental Medicine, School of Biosciences & Medicine, University of Surrey, Guildford GU2 7XH, UK; z.balkhiiarova@surrey.ac.uk; 5 Department of Endocrinology, Faculty of General Medicine, Bashkir State Medical University, 450008 Ufa, Russia; endocrinology@bashgmu.ru; 6 Department of Psychology, Middlesex University, London NW4 4BT, UK

**Keywords:** type 2 diabetes, type 2 diabetes subtypes, genetic predictors

## Abstract

**Background/Objectives**: Type 2 diabetes (T2D) is a clinically and genetically heterogeneous disease. In this study, we aimed to stratify patients with T2D from the Volga-Ural region of Eurasia into distinct subgroups based on clinical characteristics and to investigate the genetic underpinnings of these clusters. **Methods**: A total of 254 Tatar individuals with T2D and 361 ethnically matched controls were recruited. Clinical clustering was performed using k-means and hierarchical algorithms on five variables: age at diagnosis, body mass index (BMI), glycated hemoglobin (HbA1c), insulin resistance (HOMA-IR), and β-cell function (HOMA-B). Genetic association analysis was conducted using logistic regression under an additive model, adjusted for age and sex, and corrected for multiple comparisons using the Benjamini–Hochberg method. **Results**: Four distinct T2D subtypes were identified—mild age-related diabetes (MARD, *n* = 25), mild obesity-related diabetes (MOD, *n* = 72), severe insulin-resistant diabetes (SIRD, *n* = 66), and severe insulin-deficient diabetes (SIDD, *n* = 52)—each with unique clinical and comorbidity profiles. SIDD patients exhibited the highest burden of microvascular complications and lowest estimated glomerular filtration rate. Nine genetic variants showed significant associations with T2D and/or specific subtypes, including loci in genes related to neurotransmission (e.g., *HTR1B*, *CHRM5*), appetite regulation (*NPY2R*), insulin signaling (*TCF7L2*, *PTEN*), and other metabolic pathways. Some variants demonstrated subtype-specific associations, underscoring the genetic heterogeneity of T2D. **Conclusions**: Our findings support the utility of clinical clustering in uncovering biologically and clinically meaningful T2D subtypes and reveal genetic variants that may contribute to this heterogeneity. These insights may inform future precision medicine approaches for T2D diagnosis and management.

## 1. Introduction

Type 2 diabetes (T2D) is characterized by considerable phenotypic heterogeneity, as well as variability in the risk of developing diabetes-related complications. In a seminal study, Ahlqvist et al. applied a machine learning-based clustering approach to routinely measured clinical variables at the time of diagnosis—including age, glycated hemoglobin (HbA1c), body mass index (BMI), the presence of glutamic acid decarboxylase (GAD) autoantibodies, and homeostasis model assessment indices (HOMA2) for insulin resistance and β-cell function [1]. This analysis identified five distinct subgroups of individuals with diabetes, each differing in pathophysiological profiles, therapeutic responses, and susceptibility to complications [1]. These subgroups have since been robustly replicated in multiple cohorts of both European and non-European ancestry [2,3,4,5,6]. Genetic analyses have revealed partial differences in diabetes-related trait architecture across clusters, supporting the hypothesis of distinct etiological pathways among the subgroups [6,7].

The use of HOMA-IR and HOMA-B indices in patients with long-standing T2D, particularly those receiving insulin therapy, poses methodological challenges that may affect cluster allocation. Furthermore, many clinical parameters are dynamic and environmentally influenced. For instance, lifestyle changes leading to weight reduction can alter both BMI and insulin resistance indices, potentially resulting in reassignment to a different cluster over time. Currently, there is no consensus on a standardized set of clinical variables for cluster derivation, though such variables significantly influence cluster structure [5]. In contrast, genetic data offer a stable, time-invariant basis for patient stratification, enabling risk assessment independent of disease duration or clinical variation. The identification of novel clinical markers grounded in genetic risk loci may elucidate pathogenic mechanisms, enhance diagnostic precision, and inform personalized treatment strategies in T2D.

Given these considerations, we propose a clustering approach based on molecular genetic profiling. The use of genetic information allows for a consistent framework for patient classification, irrespective of temporal changes in clinical phenotype [8].

In this study, we aimed to stratify patients with type 2 diabetes (T2D) from the Volga-Ural region of Eurasia into distinct clusters based on clinical parameters, taking into account varying disease durations and treatment regimens, and to investigate the associations between these clinical clusters and genetic variants in genes related to neurotransmission, appetite and energy regulation, vasopressin signaling, insulin signaling and metabolism, non-coding RNA and epigenetic regulation, β-cell function and insulin secretion, mitochondrial function, and glucocorticoid signaling [9,10].

## 2. Materials and Methods

### 2.1. Study Group

The study was approved by the Ethics Committee of the Institute of Biochemistry and Genetics, Ufa Federal Research Centre, Russian Academy of Sciences. Written informed consent was obtained from all participants for the use of their biological material in planned research.

The study utilized DNA samples from unrelated individuals of Tatar ethnicity. The case group included 254 individuals diagnosed with type 2 diabetes (T2D) who were receiving treatment at the Diabetes Department of the Verkhneyarkeyevo Central District Hospital (Verkhneyarkeyevo village, Ilishevsky District, Republic of Bashkortostan, Russian Federation). The diagnosis of T2D was established according to the criteria of the World Health Organization (WHO) Expert Committee on Diabetes (1999) and Russian clinical guidelines [11]. Detailed descriptions of anthropometric measurements and biochemical analyses are provided in [9]. Inclusion criteria for the T2D group were: age 35 years or older; diagnosis of T2D in accordance with WHO criteria (1999–2013); and absence of clinical signs suggestive of other forms of diabetes. Information on complications such as nephropathy, retinopathy, polyneuropathy, arterial hypertension, ischaemic heart disease, and cerebrovascular disease was obtained from the clinical records. The control group consisted of 361 apparently healthy individuals. Inclusion criteria for the control group were: age 35 years or older; absence of clinical or laboratory evidence of metabolic disorders; and no family history of diabetes among relatives not involved in the study. Ethnic background (up to the third generation) and family history of diabetes were assessed through direct interviews with all prospective participants.

### 2.2. Clinical Measurements

Anthropometric measurements followed WHO guidelines [12]. Body weight and height were recorded to the nearest kilogram and centimeter, respectively, and body mass index (BMI) was calculated as weight (kg) divided by height (m^2^).

Fasting and postprandial plasma glucose levels were determined using the glucose oxidase method. Glycated hemoglobin (HbA1c) was measured by high-performance liquid chromatography using the ADAMS A1c HA-8182 analyzer (Arkray, Inc., Kyoto, Japan). Serum C-peptide concentrations were assessed by enzyme-enhanced chemiluminescence on an automated IMMULITE analyzer (DPC, Los Angeles, CA, USA) with corresponding reagents from the same manufacturer. Insulin resistance was estimated using the Homeostasis Model Assessment of Insulin Resistance (HOMA-IR), calculated by the formula: HOMA-IR = 1.5 + (C-peptide [ng/mL] × fasting glucose [mmol/L] × 18)/2800 [13]. Pancreatic β-cell function was assessed using the adjusted Homeostasis Model Assessment for β-cell function (HOMA-B), calculated by the formula: HOMA-B = (100 × C-peptide [ng/mL])/(fasting glucose [mmol/L] − 3.5) [14]. As HOMA-B and HOMA-IR may be less reliable in individuals with long-lasting T2D or those receiving insulin therapy, sensitivity analyses were conducted excluding insulin-treated participants to confirm robustness of cluster assignment. These indices provide a practical estimation of insulin resistance and β-cell function and allow comparison across individuals in the cohort, but the limitations in certain subgroups are acknowledged.

Lipid profiles, including total cholesterol (TC), triglycerides (TG), high-density lipoprotein cholesterol (HDL-C), and low-density lipoprotein cholesterol (LDL-C), were measured using standard photometric methods on an Olympus biochemical analyzer (Abbott, Hamburg, Germany) with Beckman Coulter reagent kits. The estimated glomerular filtration rate (eGFR) was assessed using the Modification of Diet in Renal Disease (MDRD) Study equation, available at http://mdrd.com.

### 2.3. Genotyping

Whole venous blood samples were collected from each participant, stored at −4 °C, and used for total DNA extraction. Genetic variants for analysis were selected based on findings from Phenome-Wide Association Studies (PheWAS) [15]. The selected variants were associated with metabolic traits (e.g., T2D, fat mass, cholesterol levels) and comorbid conditions, including inflammatory diseases and diabetes-related complications. Details of the selected SNPs are described in references [9,10,16]. Allelic discrimination was performed using real-time polymerase chain reaction (PCR) on a BioRad CFX96 system (Bio-Rad Laboratories Inc., Hercules, CA, USA) with TaqMan SNP Genotyping Assays (Thermo Fisher Scientific, Waltham, MA, USA). As part of quality control, 5% of genotyped samples were randomly selected for repeat genotyping. All repeated results were consistent with the original genotyping data.

### 2.4. Clustering Analysis

**Figure 1:**
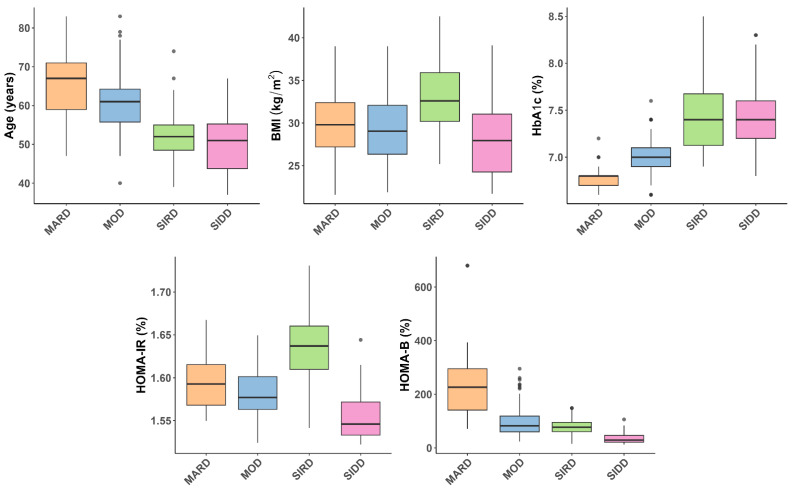
Boxplots illustrating the distribution of key clustering variables—age at diagnosis, body mass index (BMI), glycated hemoglobin (HbA1c), homeostatic model assessment insulin resistance (HOMA-IR), and homeostatic model assessment β-cell function (HOMA-B)—across the identified subgroups of type 2 diabetes: mild age-related diabetes (MARD), mild obesity-related diabetes (MOD), severe insulin resistant diabetes (SIRD), and severe insulin deficient diabetes (SIDD). Gray dots represent individual participants whose values did not reach the outlier threshold used for exclusion. Statistically significant differences between clusters, and post hoc comparisons are provided in Supplementary Table S1.

The clustering strategy employed in this study was adapted from a well-established framework that categorizes individuals with diabetes into five distinct subtypes: severe autoimmune diabetes (SAID), severe insulin-deficient diabetes (SIDD), severe insulin-resistant diabetes (SIRD), mild obesity-related diabetes (MOD), and mild age-related diabetes (MARD) [1]. As the current study did not include individuals with type 1 diabetes, the SAID cluster was not represented in our sample. Clustering was performed using a combination of k-means and hierarchical algorithms, based on five clinical variables: age at diagnosis, BMI, HbA1c, HOMA-IR, and HOMA-B [11]. Prior to clustering, 18 outliers in these variables were identified and excluded using the interquartile range (IQR) method (values below Q1 − 1.5 × IQR or above Q3 + 1.5 × IQR), to improve the stability and interpretability of the cluster structure. The exclusion of these outliers did not substantially alter the overall clustering pattern. The distribution of the key clustering variables (age, BMI, HbA1c, HOMA-IR, and HOMA-B) across the identified subgroups is illustrated in Figure 1.

Comparisons of continuous variables across clusters were conducted using either one-way ANOVA or the Kruskal–Wallis test, depending on the assumption of variance homogeneity. Levene’s test was applied to assess the equality of variances. If Levene’s test was non-significant (*p* > 0.05), ANOVA was used; otherwise, the Kruskal–Wallis test was performed. For variables with statistically significant global differences, post hoc pairwise comparisons were conducted. Tukey’s Honest Significant Difference (HSD) test was used following ANOVA, while Dunn’s test was applied following the Kruskal–Wallis test. All raw *p*-values from post hoc tests were pooled across variables and globally adjusted using the Benjamini–Hochberg procedure to control the false discovery rate (FDR) [17]. Adjusted *p*-values below 0.05 were considered statistically significant. Summary statistics for each variable include the median, first quartile (Q1), and third quartile (Q3). Categorical variables were compared between clusters using Fisher’s exact test. When the data structure precluded the use of Fisher’s test, the Chi-square test with Monte Carlo simulation for *p*-values was applied. All analyses were conducted using complete cases; no imputation was performed. Key data analysis steps, including regression modeling and summary statistics, followed established R package workflows [18]. Analyses were performed in R (version 4.4.2) using the dplyr, tidyr, car, and stats packages.

### 2.5. Association Analysis

Genetic associations with T2D, the identified T2D subtypes, and clinical parameters were evaluated using logistic or linear regression under an additive genetic model, with adjustments for age, sex, and the first two principal components from a principal component analysis (PCA) to account for population stratification. Analyses were performed in PLINK version 1.9 [19]. The additive model assumes a dose–response relationship, wherein individuals homozygous for the risk allele exhibit approximately twice the effect on the outcome compared to heterozygotes. Associations were tested across several comparisons: all T2D cases versus controls, each individual cluster versus controls, and each cluster compared to the remaining clusters both separately and collectively (e.g., Cluster 1 vs. Clusters 2, 3, and 4).

To quantify the ability of our study to detect genetic effects, we performed power calculations for the studied SNVs under an additive model using the genpwr R package [20]. These calculations incorporated the observed sample sizes, the minor allele frequencies, odds rations, and significance thresholds for each SNV. Estimated power ranged from approximately 0.62 to 0.996 (Supplementary Table S2), reflecting variation depending on allele frequency, effect size, and *p*-value. To account for multiple testing, the Benjamini–Hochberg procedure was applied to control the false discovery rate (FDR), with P_FDR_ values below 0.05 considered statistically significant.

## 3. Results

### 3.1. Clustering Analysis Results

Using a combination of k-means and hierarchical clustering algorithms, we classified a cohort of patients with T2D (*n* = 254) into four distinct subtypes based on five clinical variables: age at diagnosis, BMI, HbA1c, HOMA-IR, and HOMA-B. The resulting clusters aligned with the established diabetes subtypes: MARD, MOD, SIRD, and SIDD. Table 1 summarizes the comparison of clinical characteristics across the four identified subgroups. As expected, the clustering variables showed significant differences between subtypes, including age at diagnosis (Table 1), along with the variables used to calculate HOMA indices—fasting glucose (P_FDR_ = 2.19 × 10^−25^) and C-peptide (P_FDR_ = 2.76 × 10^−32^). Participants classified as SIDD had the longest T2D duration (P_FDR_ = 3.49 × 10^−5^) and the lowest eGFR (P_FDR_ = 0.088) compared to other subtypes. No significant differences in lipid profiles were observed across the T2D subgroups (Table 1).

Post hoc analysis revealed significant differences across most cluster pairs. Age at onset was later in MOD compared to SIRD (*p* = 6.67 × 10^−9^), and earlier in SIDD than MOD (*p* = 6.11 × 10^−11^) and MARD (*p* = 1.15 × 10^−12^) (Supplementary Table S1). MARD also had later onset than SIRD (*p* = 7.17 × 10^−11^). SIDD had a longer diabetes duration than MOD, SIRD, and MARD (all *p* < 0.014). BMI was highest in SIRD, significantly exceeding that of MOD, MARD, and SIDD (all *p* ≤ 0.00129). For glycaemic traits, fasting glucose was higher in SIRD and SIDD compared to MOD and MARD (all *p* ≤ 4.73 × 10^−5^), with no difference between SIRD and SIDD. Postprandial glucose was elevated in SIRD and SIDD versus MOD (*p* = 4.04 × 10^−10^ and 1.31 × 10^−5^), and in MARD compared to SIRD and SIDD (*p* ≤ 4.87 × 10^−4^). HbA1c was higher in SIDD and SIRD than in MOD and MARD (all *p* ≤ 2.64 × 10^−14^), with MOD also exceeding MARD (*p* = 0.00341). C-peptide levels were significantly higher in SIRD, MARD, and SIDD compared to MOD (all *p* ≤ 1.27 × 10^−5^), and SIDD differed from both SIRD and MARD (*p* < 2.2 × 10^−16^). HOMA-IR was highest in SIRD (all comparisons *p* < 1 × 10^−6^), while HOMA-B was lowest in SIDD (*p* ≤ 4.04 × 10^−10^ vs. all), and highest in MARD compared to MOD and SIRD. For renal function, the overall difference in eGFR across clusters did not reach statistical significance after FDr adjustment (P_FDR_ = 0.088); post hoc pairwise comparisons indicated that SIDD had lower eGFR than SIRD (*p* = 8.49 × 10^−4^), and SIRD had lower eGFR than MARD (*p* = 0.01), while other comparisons were not statistically significant (Supplementary Table S1). No significant differences were found between clusters for any lipid traits (Supplementary Table S1).

Significant differences in the prevalence of diabetes-related comorbidities were observed across subgroups (Table 2). The SIDD group, characterized by the lowest median age (51 years, P_FDR_ = 4.24 × 10^−19^), had the highest prevalence of retinopathy (67.31%, P_FDR_ = 5.0 × 10^−4^), nephropathy (57.69%, P_FDR_ = 0.001), and polyneuropathy (78.85%, P_FDR_ = 5.0 × 10^−4^). In contrast, the MARD group showed the highest prevalence of ischemic heart disease (64.00%, P_FDR_ = 5.0 × 10^−4^), arterial hypertension (100.00%, P_FDR_ = 0.020), and cerebrovascular disease (48.00%, P_FDR_ = 0.005). These findings are consistent with the age-related risk profile of these complications and highlight the relatively distinct clinical characteristics of the clusters.

### 3.2. Association Analysis Results

We identified nine distinct genetic variants significantly associated with T2D and/or its subtypes after correction for multiple comparisons (Table 3). Among these, *ADCY3* rs17799872 and *LINC02227* rs2149954 were associated with T2D, MOD, SIRD, and SIDD; *AVPR1B* rs33911258 and *TCF7L2* rs7903146 showed associations with T2D, MARD, MOD, and SIRD. *CHRM5* rs7162140 and *NPY2R* rs1047214 were linked to T2D, MOD, and SIRD, while *CHRNA7* rs3826029 and *PTEN* rs2735343 were associated with T2D and SIRD. *HTR1B* rs6296 was associated with T2D, MARD, and SIRD, and also remained significantly in the direct comparison between MARD and MOD. Additional loci exhibited nominal associations with T2D and/or its subtypes, but these did not withstand multiple testing correction and are listed in Supplementary Table S1.

We investigated associations between genetic variants and clinical phenotypes (Supplementary Table S2). Nominally significant associations are presented in Table 4. The *ADCY3* variant rs17799872 was associated with BMI (β = 1.26, *p* = 0.042), while *TCF7L2* rs7903146 showed associations with glycaemic traits, including postprandial glucose, HbA1c, and C-peptide levels. Variants in neuromediator receptor genes demonstrated inverse associations with lipid traits, for example, *GRIK3* rs534131 with LDL-C (β = −0.41, *p* = 0.014), *GABRA* rs279845 with triglycerides (β = −0.28, *p* = 0.018), and *CHRNA7* rs3826029 with the atherogenic coefficient (β = −0.43, *p* = 0.011), suggesting potential anti-atherogenic effects. However, none of these associations remained statistically significant after Benjamini–Hochberg correction.

**Figure 2:**
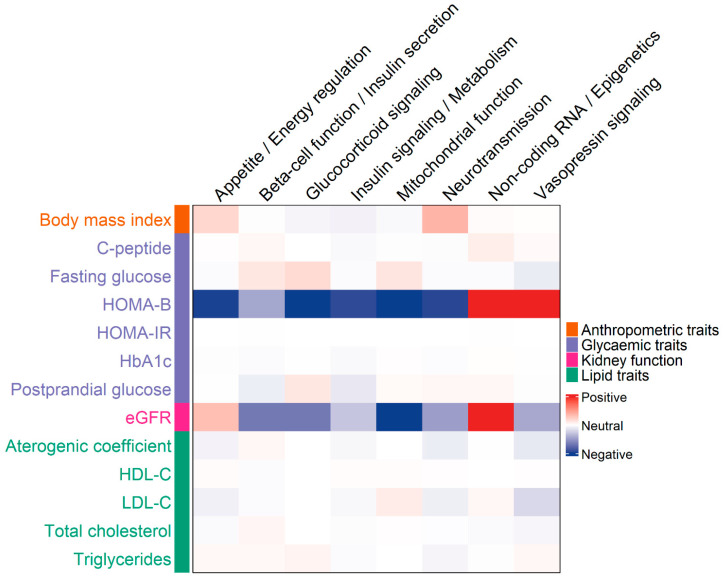
Heatmap showing associations between clinical traits and gene groups implicated in type 2 diabetes (T2D). Columns represent gene groups, and rows represent clinical parameters. The color intensity of each cell reflects the Z-score from linear regression analyses, adjusted for covariates and aligned to the effect allele. The Z-score captures both the strength and direction of the association between each gene group and clinical trait.

In our analysis of associations between clinical parameters and genetic groups linked to T2D, including appetite/energy regulation, β-cell function/insulin secretion, glucocorticoid signaling, insulin signaling/metabolism, mitochondrial function, neurotransmission, non-coding RNA/epigenetics, and vasopressin signaling, we observed pronounced differences for two key traits (Supplementary Figure S1). HOMA-B showed positive associations with the non-coding RNA and vasopressin signaling groups, while all other groups displayed negative associations. Similarly, eGFR was positively associated with variants in the non-coding RNA group and negatively associated with those involved in mitochondrial function (Supplementary Table S3, Figure 2). Other clinical parameters did not exhibit marked differences across the genetic groups.

## 4. Discussion

In this study, we successfully replicated the diabetes subtyping approach proposed by Ahlqvist et al. [1] by applying cluster analysis to clinical data from 236 individuals with T2D. Using five key variables—age at diagnosis, BMI, HbA1c, HOMA-IR, and HOMA-B—we identified four distinct clusters corresponding to previously described subtypes: mild obesity-related diabetes (MOD), severe insulin-resistant diabetes (SIRD), mild age-related diabetes (MARD), and severe insulin-deficient diabetes (SIDD). Each cluster displayed unique clinical characteristics, consistent with the underlying pathophysiological mechanisms. MOD was characterized by higher BMI, moderate insulin resistance, and relatively preserved β-cell function, indicative of metabolically milder disease despite obesity. SIRD showed the highest HOMA-IR values with relatively intact insulin secretion, pointing to severe insulin resistance as the predominant defect. The participants in the MARD cluster were older than those in the other clusters, showing mild to moderate metabolic disturbances and a less aggressive disease course. Conversely, the SIDD cluster exhibited the lowest glycemic control (elevated HbA1c) and the most severe insulin deficiency (lowest HOMA-B), suggesting a more aggressive, progressive, and potentially harder-to-manage form of diabetes. Our ability to reproduce these clusters in an independent cohort highlights the robustness and generalisability of this classification. By identifying subtypes with distinct metabolic profiles, this approach holds clinical relevance. For instance, patients with SIRD may particularly benefit from insulin-sensitizing therapies, while those in the SIDD cluster may require earlier initiation of insulin to prevent rapid disease progression. Such stratification illustrates the potential of refining therapeutic strategies by aligning treatment with distinct metabolic profiles. For example, patients with SIRD may respond better to insulin-sensitizing therapies, whereas those in the SIDD cluster may require earlier insulin initiation. However, the present findings are based on cross-sectional data and should be interpreted as hypothesis-generating rather than directly translatable to clinical decision-making. Importantly, in settings with limited healthcare resources, the feasibility of implementing clustering approaches remains uncertain, underscoring the need for studies that test whether simplified stratification can support treatment decisions in routine care.

Beyond phenotypic clustering, our study also explored genetic associations with T2D and its subtypes. Several variants showed significant associations with the overall T2D group and/or specific clusters. It should be noted that several effect sizes (ORs) appear unusually large or small (Table 3). For example, *CHRM5* rs7162140 showed an OR of 2.76 in the control vs. T2D comparison, 4.43 in control vs. MOD, and 2.77 in control vs. SIRD. Similarly, *NPY2R* rs1047214 had an OR of 2.56 in control vs. T2D, 2.63 in control vs. MOD, and 2.83 in control vs. SIRD. In contrast, *ADCY3* rs17799872 demonstrated strong protective effects, with an OR of 0.25 in the overall control vs. T2D analysis, 0.31 in control vs. MOD, 0.26 in control vs. SIRD, and 0.24 in control vs. SIDD. Given the modest sizes of both the control group (*n* = 361) and the T2D cohort (*n* = 254), and especially the smaller subgroup samples (MARD, *n* = 25; MOD, *n* = 72; SIRD, *n* = 66; SIDD, *n* = 52), these results may reflect overestimation and require replication in independent cohorts.

Notably, very high ORs could also indicate effects mediated by behavioral pathways influencing diabetes onset. *CHRM5* rs7162140, which yielded the strongest associations in our study, has previously been linked to drug addiction [21], and some authors have suggested that its association of this SNP with diabetes may be driven by its role in shaping risk behaviors that predispose to the disease [22]. *NPY2R*, in turn, encodes a that inhibits appetite regulation, thereby contributing to obesity [23].

Variants in *ADCY3* (rs17799872) and *LINC02227* (rs2149954) were associated with T2D, MOD, SIRD, and SIDD, suggesting a potential role in both general susceptibility and subtype-specific pathogenesis. Of particular interest, *ADCY3* encodes adenylate cyclase 3, which generates cyclic AMP (cAMP), a central messenger in insulin secretion, energy balance, and adipose tissue function. *ADCY3* expression is highest in adipose tissues and lower in pancreatic islets and skeletal muscle. Loss-of-function mutations in *ADCY3* have been linked to obesity and T2D in humans and mice, whereas gain-of-function mutations are protective against diet-induced obesity [24,25,26,27]. Rare and common variants at this locus are consistently associated with BMI, fat mass, and diabetes risk across populations [28,29,30,31]. Collectively, these findings support a causal role for ADCY3 in linking adiposity with glucose metabolism, potentially contributing to the pathophysiology of the MOD and SIRD subtypes.

Interestingly, syndromic forms of obesity, including Bardet-Biedl and Alström syndromes, also combine obesity with diabetes [28]. Moreover, variation in *ADCY5*, a related gene, has been associated with fasting glucose and T2D risk [29]. In humans, polymorphic variants at the *ADCY3* locus are associated with increased BMI and fat mass [30], while carriers of loss-of-function alleles present with obesity, insulin resistance, dyslipidemia, and T2D. Reduced *ADCY3* RNA expression in carriers further supports a loss-of-function mechanism [31]. The association of rare *ADCY3* variants with T2D has also been replicated in across populations, underscoring its relevance for both disease risk and therapy development [30]. Recent findings point to a critical role of *ADCY3* signaling in neuronal cilia, providing new mechanistic insight into obesity susceptibility [31].

*AVPR1B* (rs33911258) and *TCF7L2* (rs7903146), known for their roles in glycemic regulation and β-cell function, were associated with T2D, MARD, MOD, and SIRD, underscoring their broad involvement in metabolic phenotypes. *TCF7L2* is among the strongest and most consistently replicated loci for T2D, first identified through both linkage [32] and genome-wide association studies (GWAS) [33]. Its association has been confirmed across diverse populations, representing one of the most robust genetic findings for a complex disease. Meta-analyses indicate that *TCF7L2* variants act under a multiplicative model and may contribute to up to 20% of T2D cases [34]. The mechanisms remain incompletely defined, but TCF7L2 is a key component of the Wnt signaling pathway, regulating transcriptional programs central to glucose and lipid metabolism. It shapes adipocyte size, endocrine function, and glucose homeostasis, and its expression is reduced in white adipose tissue under diet-induced obesity [35]. Genome-wide profiling shows that TCF7L2 directly regulates genes controlling metabolism and cell cycle progression. In animal models, adipocyte-specific deletion of *Tcf7l2* combined with high-fat diet leads to early-onset glucose intolerance, insulin resistance, weight gain, and adipose expansion, with glucose dysregulation evident by day 3 and insulin resistance by 3 months [36]. These findings suggest that impaired *TCF7L2* function heightens susceptibility to diabetes under metabolic stress.

Neuropeptide Y (NPY) is a sympathetic neurotransmitter preferentially released during sustained stress, where it drives vasoconstriction and vascular smooth muscle proliferation. The neuropeptide Y receptor type 2 (NPY2R), which responds to NPY, is involved in angiogenesis and energy homeostasis. A recent phase I trial of the novel NPY2R agonist BI 1820237 demonstrated good tolerability and potential for weight modulation, both as monotherapy and in combination with low-dose liraglutide in overweight or obese individuals [37]. NPY and peptide YY (PYY) belong to the same peptide family and bind Y-type G protein coupled receptors (GPCRs) in the central nervous system to regulate appetite and feeding behavior, making them attractive targets for weight management therapies [38]. Clinical studies show that PYY isoforms (PYY1-36, PYY3-36) modulate appetite, energy intake, and metabolism in both lean and obese individuals [30,39]. Preclinical work further supports the therapeutic potential of NPY2R agonists: a modified PYY peptide reduced food intake in mice by suppressing orexigenic NPY/AgRP neuron activity [40].

Arginine vasopressin (AVP) is traditionally recognized for its role in fluid homeostasis, but emerging evidence indicates effects on pancreatic β-cell function. Its receptors (Avpr1a, Avpr1b, Avpr2) are expressed in rodent and human β-cells [41]. AVP stimulates insulin secretion, promotes βcell proliferation, and protects against cytokine-induced apoptosis, without affecting glucagon [42]. In vivo, AVP administration with glucose lowers blood glucose while increasing plasma insulin. These results reveal previously underappreciated endocrine roles for AVP and suggest that receptor signaling in islets may contribute to β-cell dysfunction in diabetes.

Variants in *CHRM5* (rs7162140), *CHRNA7* (rs3826029), and *HTR1B* (rs6296), were associated with T2D and specific subtypes in our cohort, implicating neurotransmitter pathways in metabolic heterogeneity. *CHRM5* rs7162140, the only common polymorphism in the locus s (minor allele frequency 9% overall; 21.7% in Europeans), has been linked to addictive behaviors that may increase cardiometabolic risk [21,22], and in our study was associated with T2D, SIRD, and MOD, suggesting contributions via metabolic and behavioral pathways. *CHRNA7* rs3826029, a promoter variant previously linked to bipolar disorder [43], showed a protective association with T2D, and particularly SIRD subtype, indicating a potentially distinct mechanism *HTR1B* rs6296, a synonymous variant reducing gene expression and previously linked to psychiatric and behavioral traits [44,45,46,47,48], was inversely associated with T2D overall, with stronger effects for SIRD and MARD, and distinguished MARD from MOD. These findings support a role for serotonin and cholinergic signaling, potentially interacting with dopaminergic pathways, in modulating both behavioral traits and T2D susceptibility.

While our study primarily addresses genetic heterogeneity, T2D arises from a complex interplay between inherited susceptibility and environmental influences. Lifestyle-related risk factors such as diet, physical activity, and obesity remain well-established contributors, but emerging evidence also points to the role of broader environmental exposures. For example, a recent nationwide cross-sectional study reported associations between bottled water consumption and chronic disease risk, including diabetes [49]. Considering such findings underscores the importance of integrating genetic, environmental, and lifestyle perspectives in understanding disease etiology. Situating our results within this multifactorial framework highlights that genetic heterogeneity should not be interpreted in isolation but rather as one component within a broader network of determinants of T2D.

### Study Strengths and Limitations

Despite the promising insights, our study has several limitations. Although the overall sample comprised 615 individuals, the relatively modest number of T2D cases (*n* = 254) subdivided into four clusters may affect the robustness of the clustering results and limit the statistical power to detect subtype-specific genetic associations. Power calculations for the studied SNVs under an additive genetic model at the observed significance thresholds showed estimated power ranging from approximately 0.62 to 0.996, indicating that while many larger-effect variants could be detected, variants with smaller effect sizes or lower minor allele frequencies remain underpowered. This limitation is particularly evident for the smallest subgroup, MARD (*n* = 25), where results should be interpreted cautiously. Our study is also limited by its cross-sectional design, which precludes causal inference. Differences in complications across clusters cannot be interpreted as evidence that specific subtypes, such as SIDD, directly predispose to microvascular or other complications, as longer disease duration may contribute to these outcomes. Longitudinal studies are needed to clarify temporal relationships and establish causality. Nevertheless, the clear phenotypic differentiation and concordance with previously reported clusters support the validity of our findings. Larger cohorts and longitudinal follow-up will be essential to validate these findings, improve the robustness of subtype-specific analyses, and explore their implications for disease progression and treatment response.

## 5. Conclusions

Using a validated clustering approach, we replicated the four main type 2 diabetes (T2D) subtypes—MARD, MOD, SIRD, and SIDD—in our cohort, each displaying distinct clinical profiles. SIDD was the most metabolically severe, with low glycemic control and the highest burden of microvascular complications. MARD, associated with older age at diagnosis, showed the greater prevalence of macrovascular disease. SIRD and MOD were characterized by insulin resistance and obesity, with MOD showing earlier onset. We identified nine genetic variants associated with T2D and/or specific subtypes, including *ADCY3*, *TCF7L2*, and *CHRNA7*, highlighting genetic heterogeneity across clusters. Notably, the associations of *ADCY3 rs17799872* with MOD, SIRD, and SIDD suggest a shared genetic basis for obesity and glycemic dysregulation. While phenotypic lipid profiles did not differ across clusters, nominal associations with lipid-related genes (e.g., *CHRNA7*, *GRIK3*) suggest subtype-specific metabolic mechanisms. Pathway-based analyses further supported biological differentiation: vasopressin and non-coding RNA pathways were linked to β-cell function, and mitochondrial pathways to kidney function. These findings underscore the clinical and genetic diversity within T2D. Empirically, we replicated the four established subtypes and identified several genetic variants associated with T2D and/or specific clusters highlighting heterogeneity at both the clinical and genetic level. While such evidence supports the potential utility of integrated clustering and genetic approaches to inform precision medicine, translation to treatment remains speculative. Future multi-ethnic, longitudinal studies will be essential to determine whether subtype-based stratification can improve therapeutic outcomes and how such approaches might be adapted for different healthcare settings, including those with limited resources.

## Figures and Tables

**Table 1:** Clinical characteristics of the clusters.

Parameter	MARD (*n* = 25)	MOD (*n* = 72)	SIRD (*n* = 66)	SIDD (*n* = 52)	^1^ *p*	^2^ P_FDR_
Age (years)	67 (59–71)	61 (55.75–64.25)	52 (48.5–55)	51 (43.75–55.25)	1.69 × 10^−19^	4.24 × 10^−19^
T2D duration (years)	5 (1–8)	3 (1–7)	4 (1.25–6)	8 (4–14)	2.10 × 10^−5^	3.49×10^−5^
^3^ BMI (kg/m^2^)	29.8 (27.2–32.4)	29.05 (26.35–32.08)	32.6 (30.2–35.9)	27.95 (24.28–31.05)	1.42 × 10^−9^	2.66×10^−9^
Fasting glucose (mmol/L)	4.9 (4.3–5.8)	6.1 (5.57–6.5)	7.3 (6.73–7.97)	7.15 (6.68–7.3)	4.37 × 10^−26^	2.19×10^−25^
Postprandial glucose (mmol/L)	7.8 (7.8–8.3)	8.4 (7.8–8.9)	10.3 (9.95–10.8)	9.95 (9.3–10.38)	8.51 × 10^−13^	1.82×10^−12^
^4^ HbA1c (%)	6.8 (6.7–6.8)	7 (6.9–7.1)	7.4 (7.12–7.68)	7.4 (7.2–7.6)	2.01 × 10^−21^	6.02×10^−21^
C peptide (ng/mL)	3.1 (2.4–3.4)	1.9 (1.64–2.61)	3 (2.48–3.39)	1 (0.72–1.61)	1.84 × 10^−33^	2.76×10^−32^
HOMA-IR (%)	1.59 (1.57–1.62)	1.58 (1.56–1.6)	1.64 (1.61–1.66)	1.55 (1.53–1.57)	8.39 × 10^−33^	6.29×10^−32^
HOMA-B (%)	226.67 (141.3–295.71)	82.45 (60.3–118.93)	77.25 (60.67–95.62)	29.22 (21.52–46.63)	6.79 × 10^−24^	2.54×10^−23^
Total cholesterol (mmol/L)	4.9 (4.2–6.1)	5.4 (4.7–6)	5.3 (4.62–6.1)	5.3 (4.5–6.55)	0.528	0.61
Triglycerides (mmol/L)	1.43 (0.89–1.8)	1.32 (0.98–2)	1.31 (1.01–2.13)	1.4 (0.9–1.84)	0.857	0.857
^5^ HDL-C (mmol/L)	1.1 (0.87–1.4)	1.2 (0.88–1.42)	1.1 (0.8–1.46)	1.2 (0.94–1.5)	0.846	0.857
^6^ LDL-C (mmol/L)	3.1 (2.2–3.79)	2.42 (1.92–3.79)	2.42 (1.8–4.52)	3.33 (2.11–4.57)	0.241	0.301
^7^ eGFR (mL/min/1.73 m^2^)	63 (56–69)	66.5 (56–77)	75 (59–92)	61.5 (53–75)	1.49 × 10^−4^	0.088

Data are presented as median (interquartile range: Q1–Q3). ^1^ *p*—significance levels based on ANOVA or Kruskal–Wallis tests, depending on variance homogeneity; ^2^ P_FDR_—significance level with false discovery rate (FDR) correction; ^3^ BMI—body mass index; ^4^ HbA1c—glycated hemoglobin; ^5^ HDL-C—high-density lipoprotein cholesterol; ^6^ LDL-C—low-density lipoprotein cholesterol; ^7^ eGFR—estimated glomerular filtration rate.

**Table 2:** Prevalence of diabetes-related comorbidities across subgroups.

Comorbidity	MARD (*n* = 25)	MOD (*n* = 72)	SIRD (*n* = 66)	SIDD (*n* = 52)	^1^ *p*	P_FDR_
Retinopathy	36.00 ± 9.60	33.33 ± 5.56	31.82 ± 5.73	67.31 ± 6.51	5.00 × 10^−4^	0.001
Nephropathy	36.00 ± 9.60	25.00 ± 5.10	25.76 ± 5.38	57.69 ± 6.85	5.00 × 10^−4^	0.001
Polyneuropathy	56.00 ± 9.93	38.89 ± 5.75	31.82 ± 5.73	78.85 ± 5.66	5.00 × 10^−4^	0.001
Ischemic heart disease	64.00 ± 9.60	41.67 ± 5.81	24.24 ± 5.28	34.62 ± 6.60	5.00 × 10^−3^	0.006
Arterial hypertension	100.00 ± 0.00	86.11 ± 4.08	74.24 ± 5.38	86.54 ± 4.73	1.95 × 10^−2^	0.020
Cerebrovascular disease	48.00 ± 9.99	36.11 ± 5.66	15.15 ± 4.41	23.08 ± 5.84	3.00 × 10^−3^	0.005

Data are presented as percentages ± standard error (SE). ^1^ *p*—significance level based on Fisher’s exact test or Chi-square test, as appropriate.

**Table 3:** Statistically significant associations between genetic variants and T2D or its subtypes after multiple comparisons correction.

Compared Groups	Gene	^1^ SNV	^2^ EA	^3^ OR (^4^ 95% CI)	^5^ *p*	^6^ P_FDR_
Control vs. T2D	*ADCY3*	rs17799872	A	0.25 (0.16–0.39)	2.79 × 10^−9^	1.12 × 10^−7^
	*CHRM5*	rs7162140	T	2.76 (1.87–4.05)	2.49 × 10^−7^	4.97 × 10^−6^
	*NPY2R*	rs1047214	C	2.56 (1.78–3.69)	4.67 × 10^−7^	6.22 × 10^−6^
	*HTR1B*	rs6296	C	0.49 (0.36–0.67)	6.20 × 10^−6^	5.86 × 10^−5^
	*LINC02227*	rs2149954	A	0.42 (0.29–0.62)	7.33 × 10^−6^	5.86 × 10^−5^
	*AVPR1B*	rs33911258	C	0.42 (0.28–0.63)	2.88 × 10^−5^	1.92 × 10^−4^
	*TCF7L2*	rs7903146	T	0.53 (0.39–0.71)	4.16 × 10^−5^	2.38 × 10^−4^
	*PTEN*	rs2735343	C	0.47 (0.32–0.68)	8.20 × 10^−5^	4.10 × 10^−4^
	*CHRNA7*	rs3826029	A	0.51 (0.34–0.76)	9.90 × 10^−4^	0.004
Control vs. MARD	*HTR1B*	rs6296	C	0.10 (0.03–0.27)	9.36 × 10^−6^	3.74 × 10^−4^
	*AVPR1B*	rs33911258	C	0.14 (0.05–0.41)	3.09 × 10^−4^	0.006
	*TCF7L2*	rs7903146	T	0.27 (0.12–0.60)	1.25 × 10^−3^	0.017
Control vs. MOD	*CHRM5*	rs7162140	T	4.43 (2.23–8.81)	2.15 × 10^−5^	0.001
	*ADCY3*	rs17799872	A	0.31 (0.17–0.59)	3.00 × 10^−4^	0.006
	*AVPR1B*	rs33911258	C	0.36 (0.20–0.64)	4.47 × 10^−4^	0.006
	*NPY2R*	rs1047214	C	2.63 (1.51–4.60)	6.92 × 10^−4^	0.006
	*TCF7L2*	rs7903146	T	0.44 (0.27–0.71)	7.42 × 10^−4^	0.006
	*LINC02227*	rs2149954	A	0.42 (0.25–0.73)	1.95 × 10^−3^	0.013
Control vs. SIRD	*ADCY3*	rs17799872	A	0.26 (0.15–0.48)	9.55 × 10^−6^	3.82 × 10^−4^
	*HTR1B*	rs6296	C	0.42 (0.28–0.64)	4.33 × 10^−5^	7.68 × 10^−4^
	*TCF7L2*	rs7903146	T	0.41 (0.27–0.63)	5.76 × 10^−5^	7.68 × 10^−4^
	*NPY2R*	rs1047214	C	2.83 (1.64–4.87)	1.79 × 10^−4^	0.002
	*LINC02227*	rs2149954	A	0.40 (0.24–0.66)	3.38 × 10^−4^	0.003
	*CHRM5*	rs7162140	T	2.77 (1.57–4.86)	4.17 × 10^−4^	0.003
	*PTEN*	rs2735343	C	0.42 (0.26–0.69)	5.03 × 10^−4^	0.003
	*CHRNA7*	rs3826029	A	0.40 (0.23–0.70)	1.09 × 10^−3^	0.005
	*AVPR1B*	rs33911258	C	0.42 (0.24–0.72)	1.71 × 10^−3^	0.008
Control vs. SIDD	*ADCY3*	rs17799872	A	0.24 (0.13–0.46)	1.55 × 10^−5^	5.57 × 10^−4^
	*LINC02227*	rs2149954	A	0.30 (0.17–0.53)	2.79 × 10^−5^	5.57 × 10^−4^
MOD vs. MARD	*HTR1B*	rs6296	C	0.31 (0.15–0.62)	1.06 × 10^−3^	0.042

^1^ SNV—single-nucleotide variant; ^2^ EA—effect allele; ^3^ OR—odds ratio; ^4^ 95%CI—95% confidence interval; ^5^ *p*—unadjusted significance level; ^6^ P_FDR_—significance level with false discovery rate (FDR) correction.

**Table 4:** Nominally significant associations between genetic variants and clinical traits in T2D patients.

Trait	Gene	^1^ SNV	^2^ RA	Beta ± ^3^ SE	^4^ 95% CI	^5^ *p*	^6^ P_FDR_
Body mass index	*ADCY3*	rs17799872	G	1.26 ± 0.62	0.05–2.47	0.042	0.561
	*HTR2A*	rs6313	A	1.04 ± 0.43	0.19–1.88	0.017	0.341
	*HTR2C*	rs6318	G	1.3 ± 0.5	0.31–2.29	0.011	0.341
Fasting glucose	*PTEN*	rs2735343	G	0.37 ± 0.16	0.05–0.69	0.023	0.715
	*GABRA*	rs279845	T	−0.24 ± 0.11	−0.46–−0.02	0.036	0.715
Postprandial glucose	*BDNF*	rs11030107	A	−0.49 ± 0.24	−0.97–−0.02	0.042	0.845
	*TCF7L2*	rs7903146	C	−0.35 ± 0.16	−0.65–−0.04	0.026	0.845
^7^ HbA1c	*LINC02227*	rs2149954	G	0.09 ± 0.05	0–0.18	0.049	0.577
	*GABRA*	rs279845	T	−0.08 ± 0.04	−0.16–−0.01	0.037	0.577
	*TCF7L2*	rs7903146	C	−0.08 ± 0.04	−0.15–−0.01	0.024	0.577
C-peptide	*LRP5*	rs3736228	C	−0.29 ± 0.15	−0.58–0	0.048	0.633
	*CDKN2BAS1*	rs4977574	G	0.22 ± 0.09	0.05–0.4	0.014	0.566
	*TCF7L2*	rs7903146	C	0.18 ± 0.08	0.01–0.34	0.038	0.633
HOMA-B	*LINC00305*	rs2850711	A	18.96 ± 9.5	0.35–37.58	0.047	0.628
HOMA-IR	*CDKN2BAS1*	rs4977574	G	0.01 ± 0	0–0.02	0.022	0.698
^8^ LDL-C	*GRIK3*	rs534131	G	−0.41 ± 0.17	−0.73–−0.09	0.014	0.542
Triglycerides	*GABRA*	rs279845	T	−0.28 ± 0.12	−0.5–−0.05	0.018	0.71
Aterogenic coefficient	*CHRNA7*	rs3826029	G	−0.43 ± 0.17	−0.76–−0.1	0.011	0.433
^9^ eGFR	*LINC02227*	rs2149954	G	−4.24 ± 1.78	−7.72–−0.76	0.018	0.375
	*CDKN2BAS1*	rs4977574	G	3.18 ± 1.51	0.22–6.14	0.036	0.386
	*MALAT1*	rs619586	A	6.92 ± 2.92	1.2–12.63	0.019	0.375
	*HTR2A*	rs6313	A	−3.11 ± 1.55	−6.14–−0.07	0.047	0.386

^1^ SNV—single-nucleotide variant; ^2^ RA—reference allele; ^3^ SE—standard error; ^4^ 95%CI—95% confidence interval; ^5^ *p*—unadjusted significance level; ^6^ P_FDR_—significance level with false discovery rate (FDR) correction, ^7^ HbA1c—glycated hemoglobin, ^8^ LDL-C—low-density lipoprotein cholesterol; ^9^ eGFR—estimated glomerular filtration rate.

## Data Availability

Data are contained within the article and Supplementary Materials.

## Supplementary Materials

The following supporting information can be downloaded at: https://www.mdpi.com/article/10.3390/genes16101131/s1, Table S1: Comparison of clinical characteristics across subtypes with post hoc analysis; Table S2: SNP associations with type 2 diabetes and its distinct clinical subtypes; Table S3: Associations between genetic variants and clinical phenotypes in individuals with type 2 diabetes; Figure S1: The results of genome-wide association studies with phenotypic traits for the variants included in this analysis.
