# Supplementary material for: Exploring Genetic Heterogeneity in Type 2 Diabetes Subtypes
Source: Genes (Basel). 2025 Sep 25;16(10):1131. doi: 10.3390/genes16101131 (PMC12564368; doi:10.3390/genes16101131)
Supplement: Supplementary file 1 [file genes-16-01131-s001.zip › genes-3803334-supplementary/genes-3803334-supplementary-proofdone/Supplementary_Figure_S1[79].pdf]

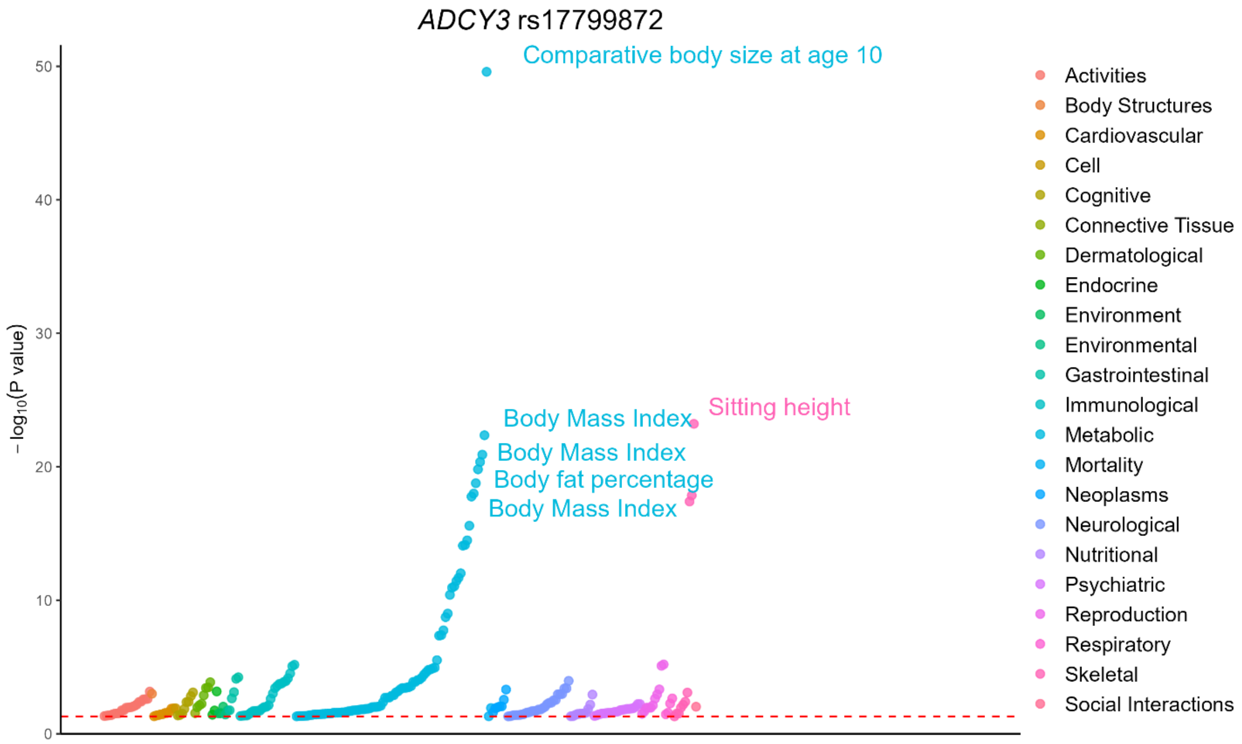

The results of the genome-wide association studies with phenotypic traits for the *ADCY3 rs17799872* variant

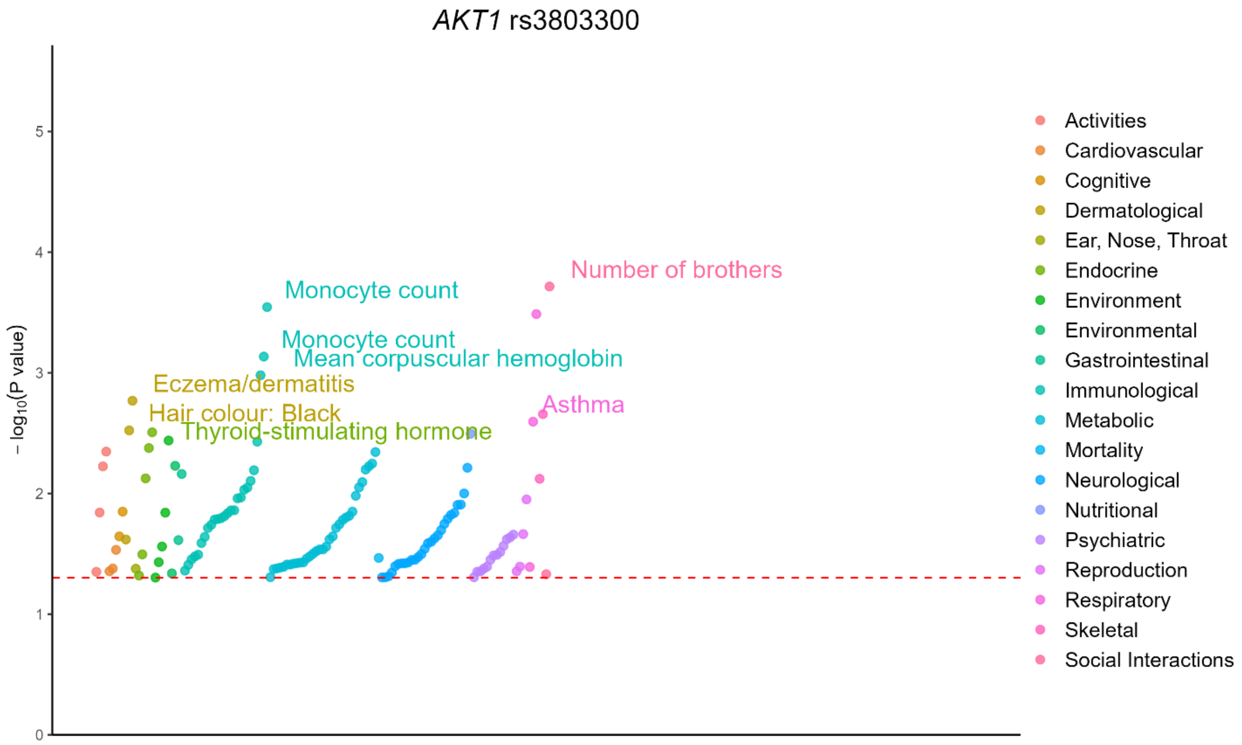

The results of the genome-wide association studies with phenotypic traits for the *AKT1 rs3803300* variant

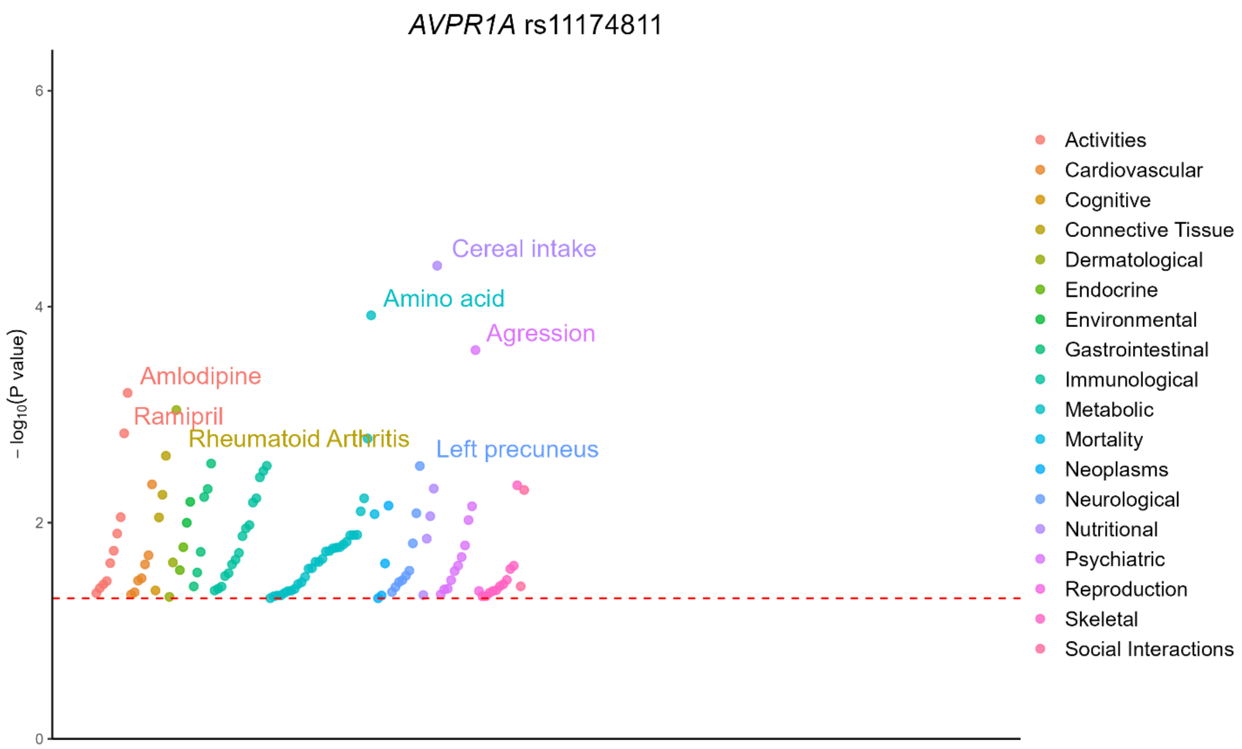

The results of the genome-wide association studies with phenotypic traits for the *AVPR1A rs11174811* variant

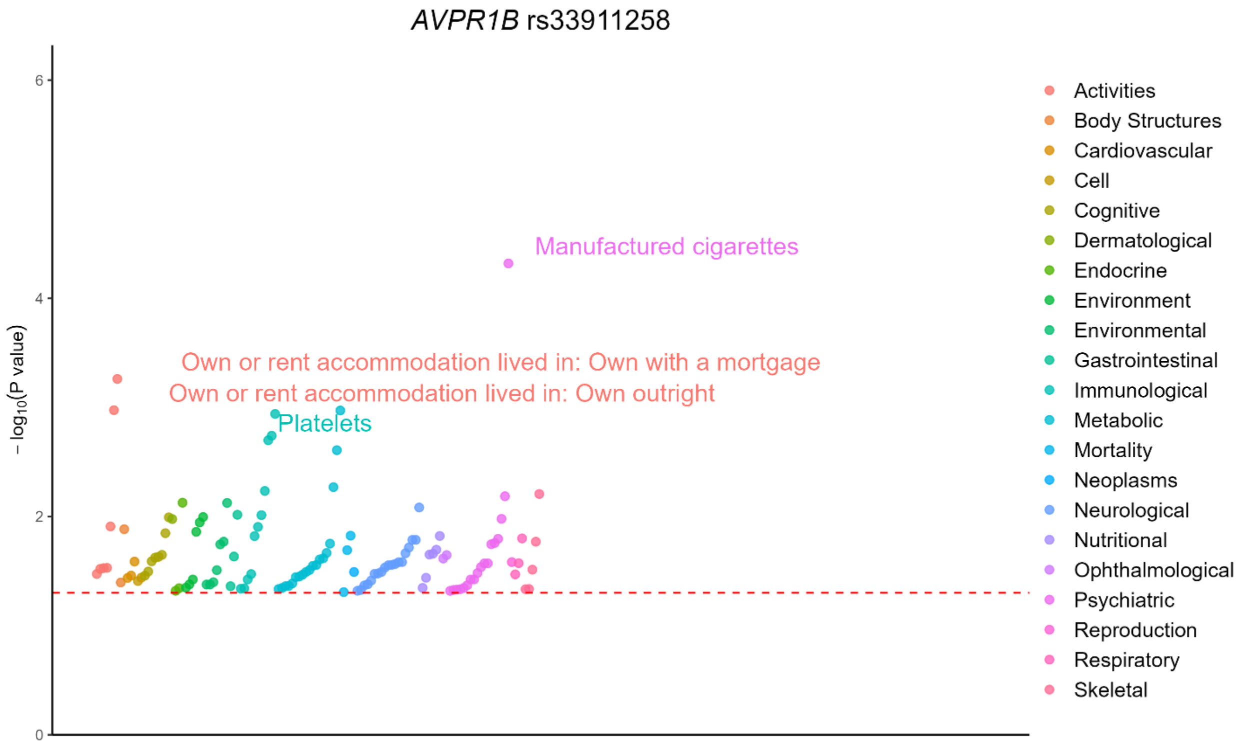

The results of the genome-wide association studies with phenotypic traits for the *AVPR1B rs33911258* variant

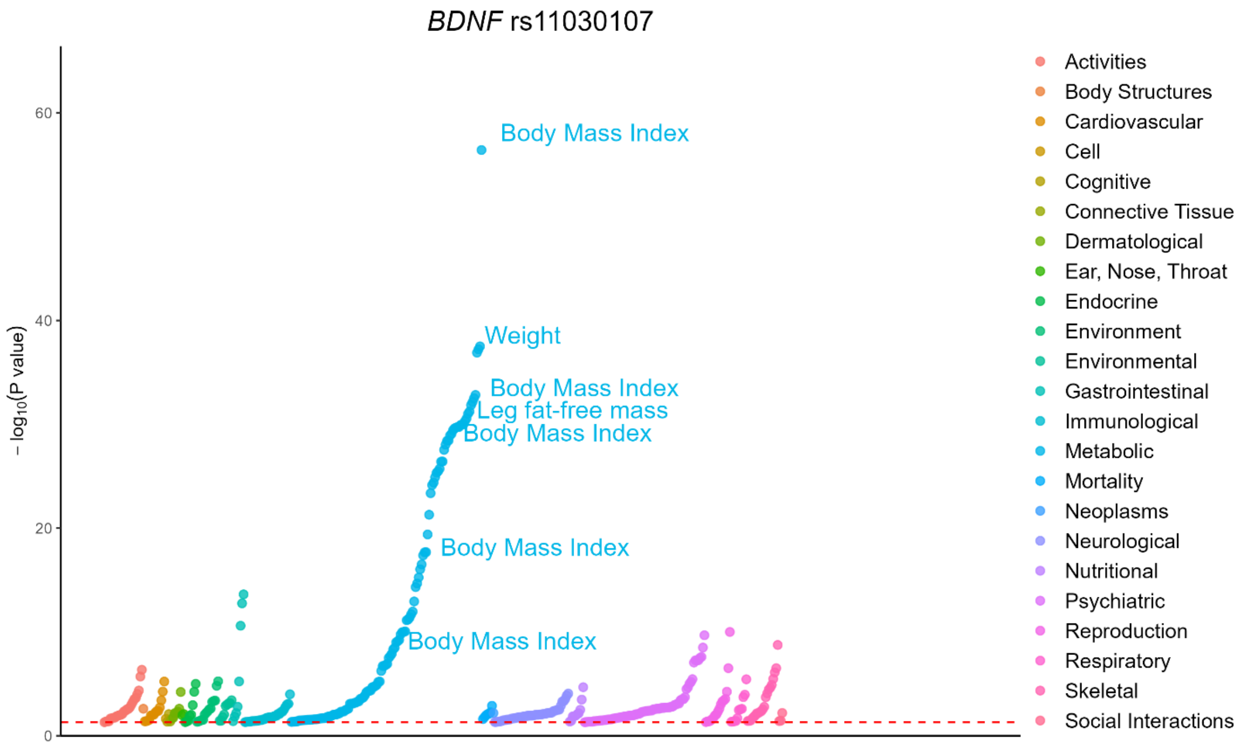

The results of the genome-wide association studies with phenotypic traits for the *BDNF rs11030107* variant

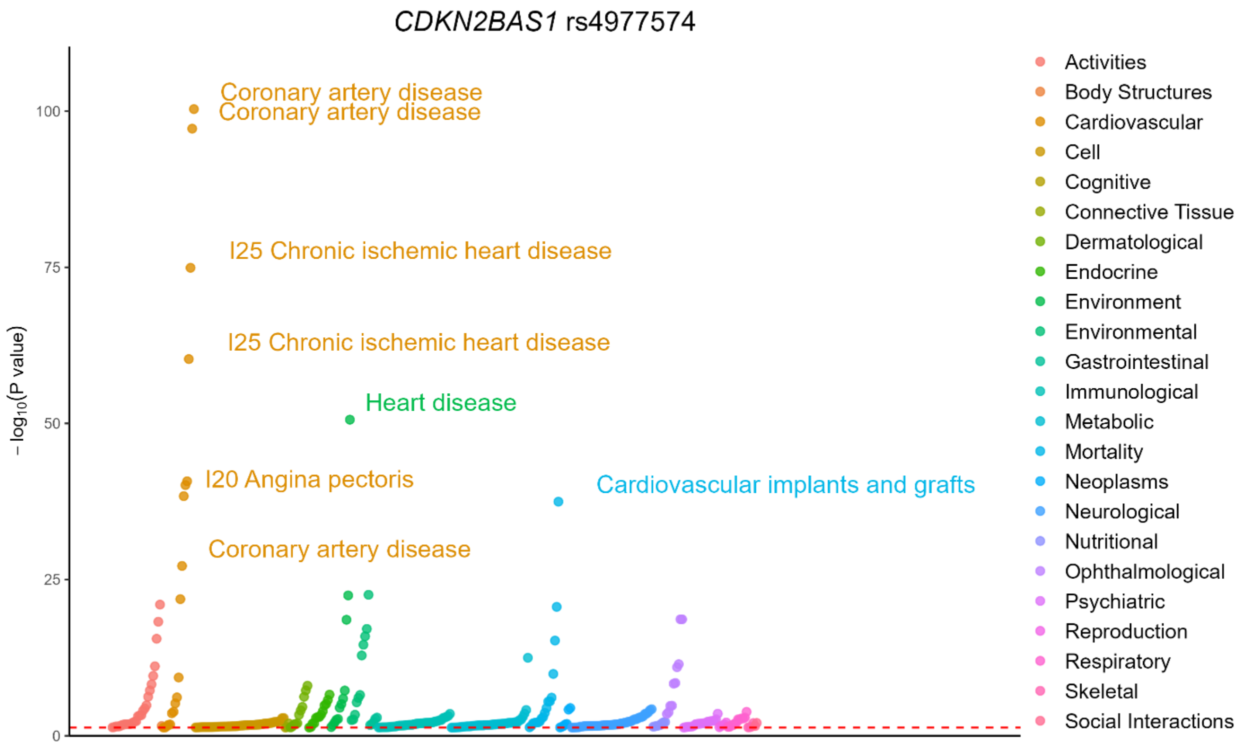

The results of the genome-wide association studies with phenotypic traits for the *CDKN2BAS1 rs4977574* variant

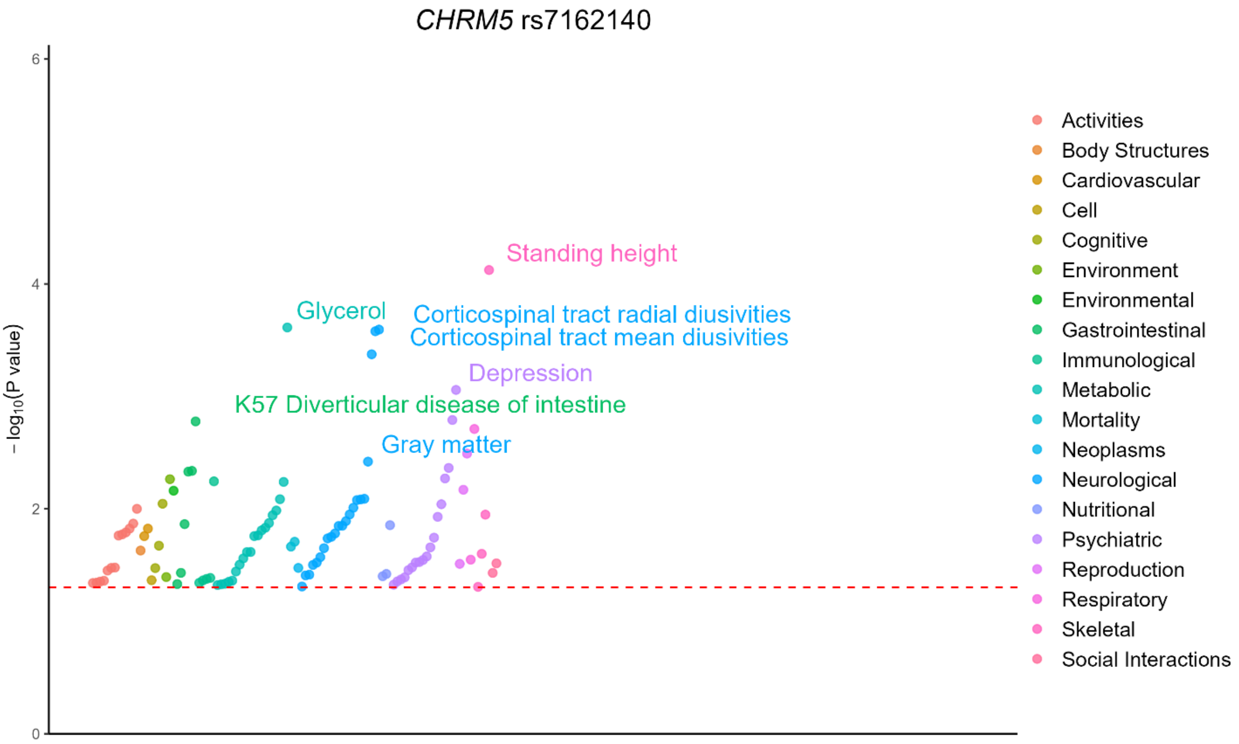

The results of the genome-wide association studies with phenotypic traits for the *CHRM5 rs7162140* variant

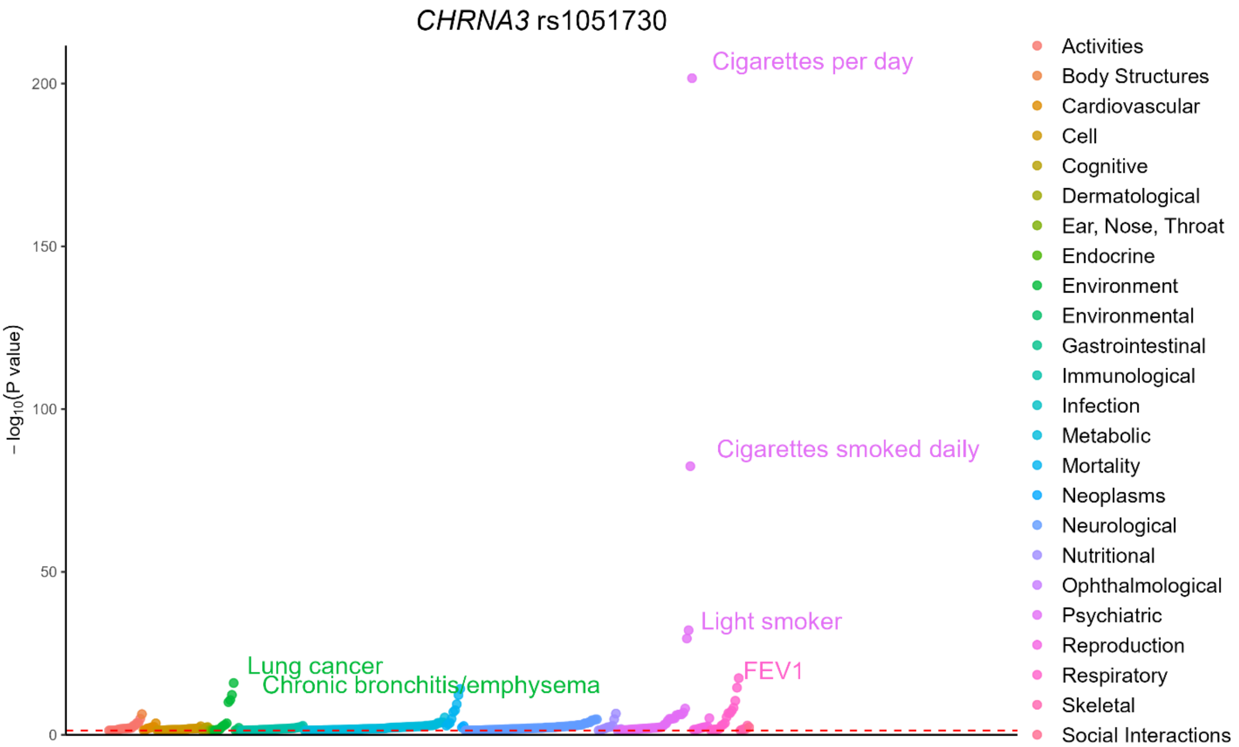

The results of the genome-wide association studies with phenotypic traits for the *CHRNA3 rs1051730* variant

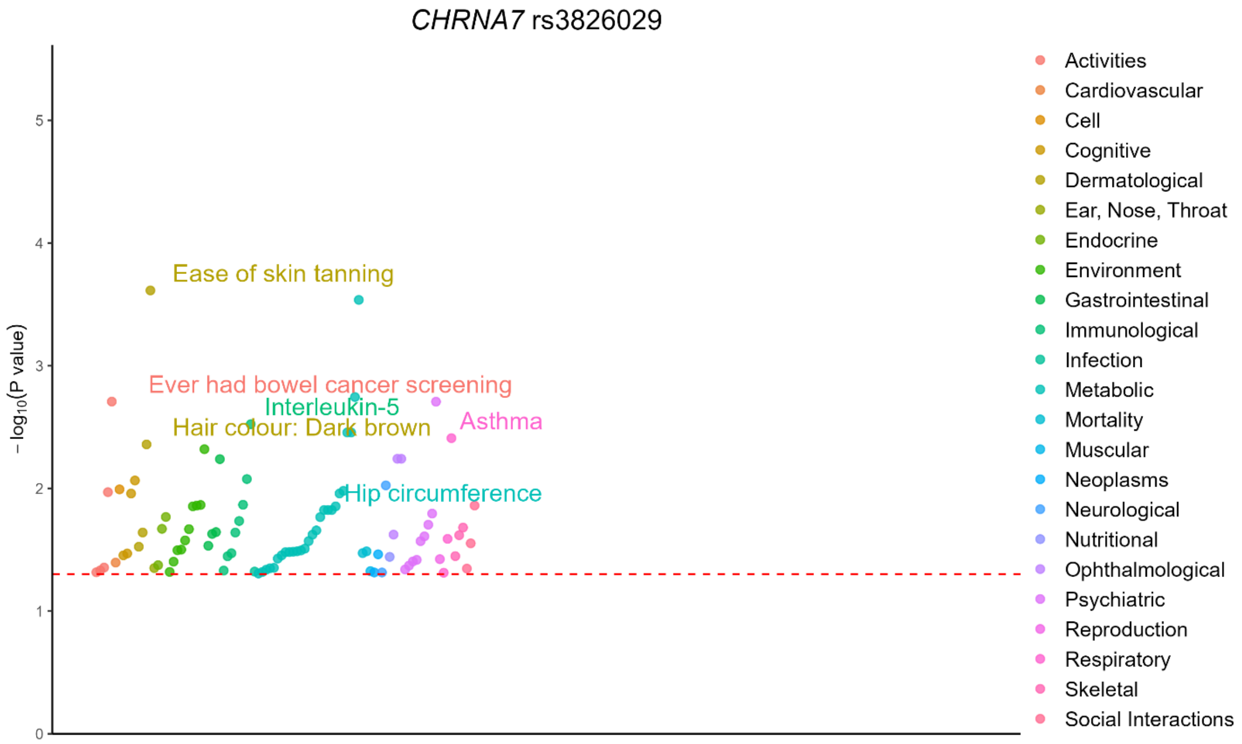

The results of the genome-wide association studies with phenotypic traits for the *CHRNA7 rs3826029* variant

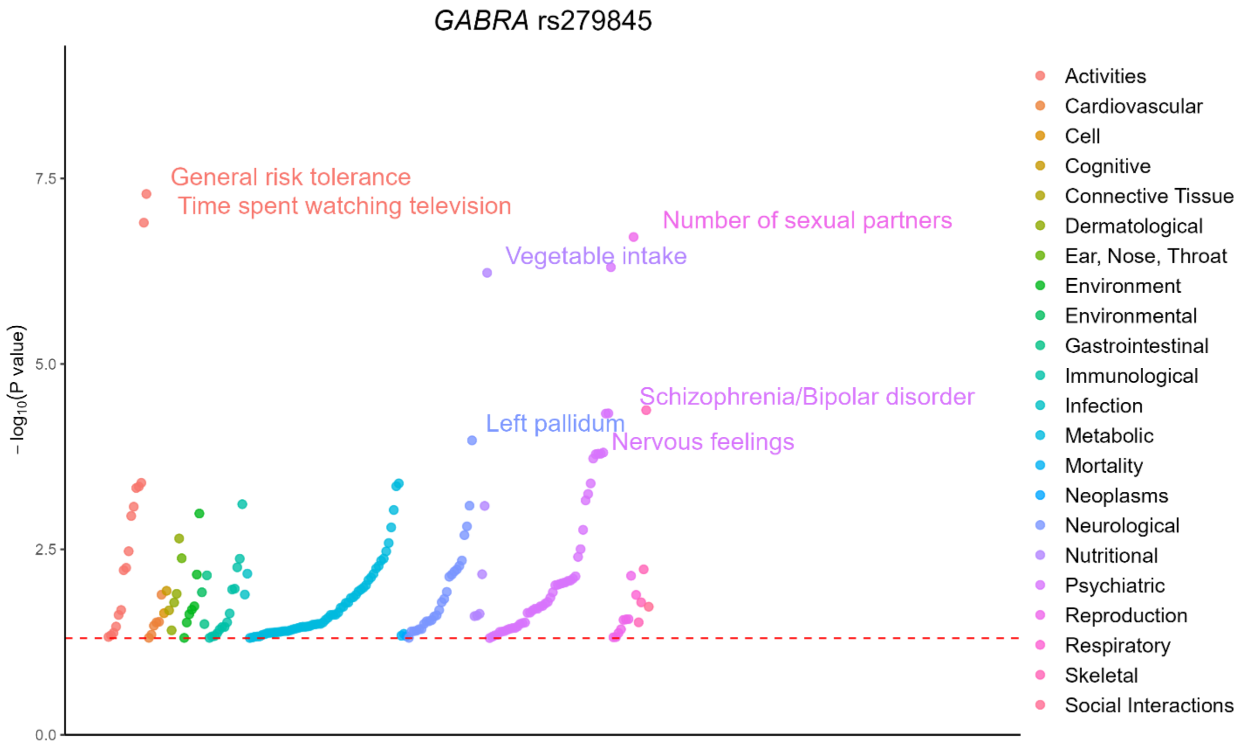

The results of the genome-wide association studies with phenotypic traits for the *GABRA rs279845* variant

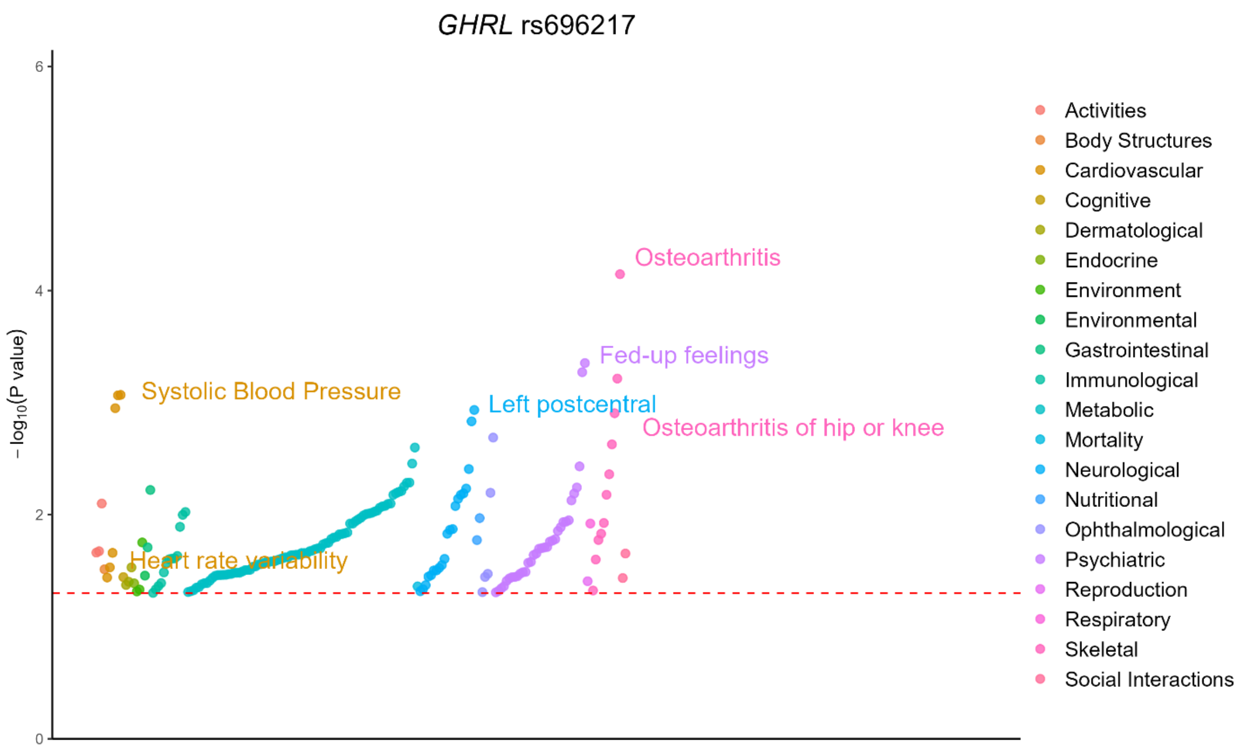

The results of the genome-wide association studies with phenotypic traits for the *GHRL rs696217* variant

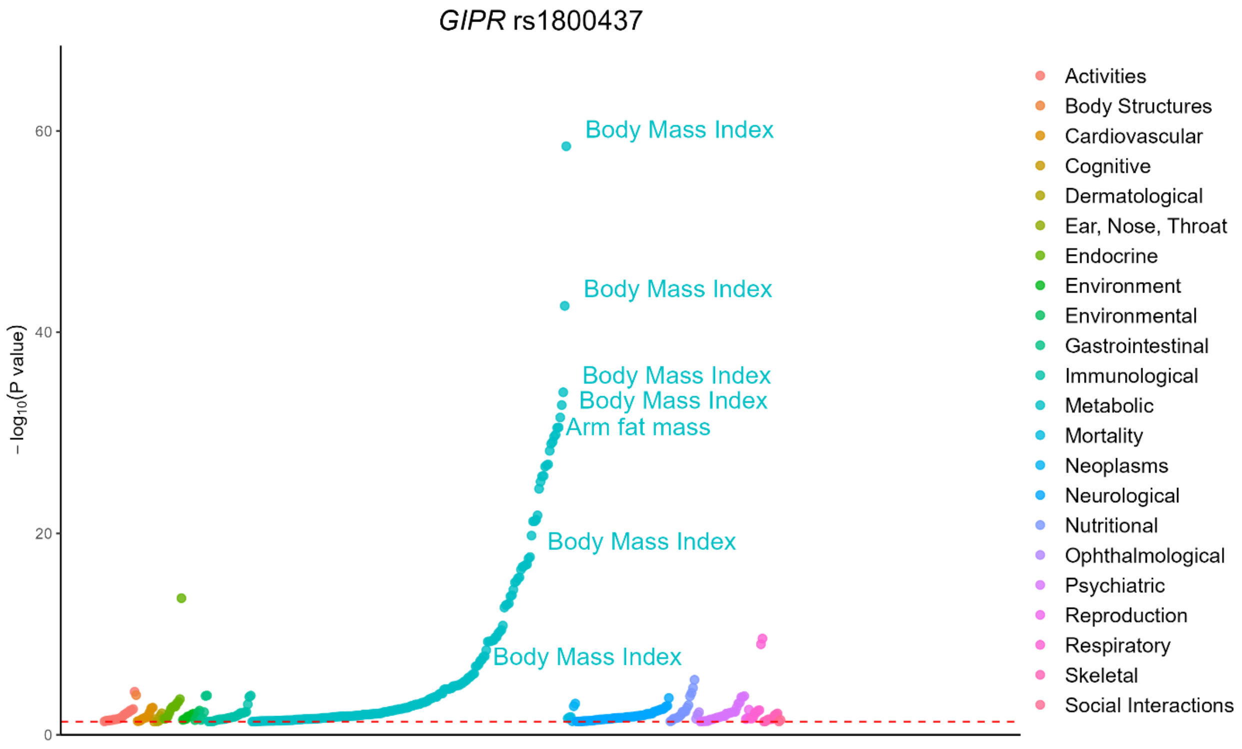

The results of the genome-wide association studies with phenotypic traits for the *GIPR rs1800437* variant

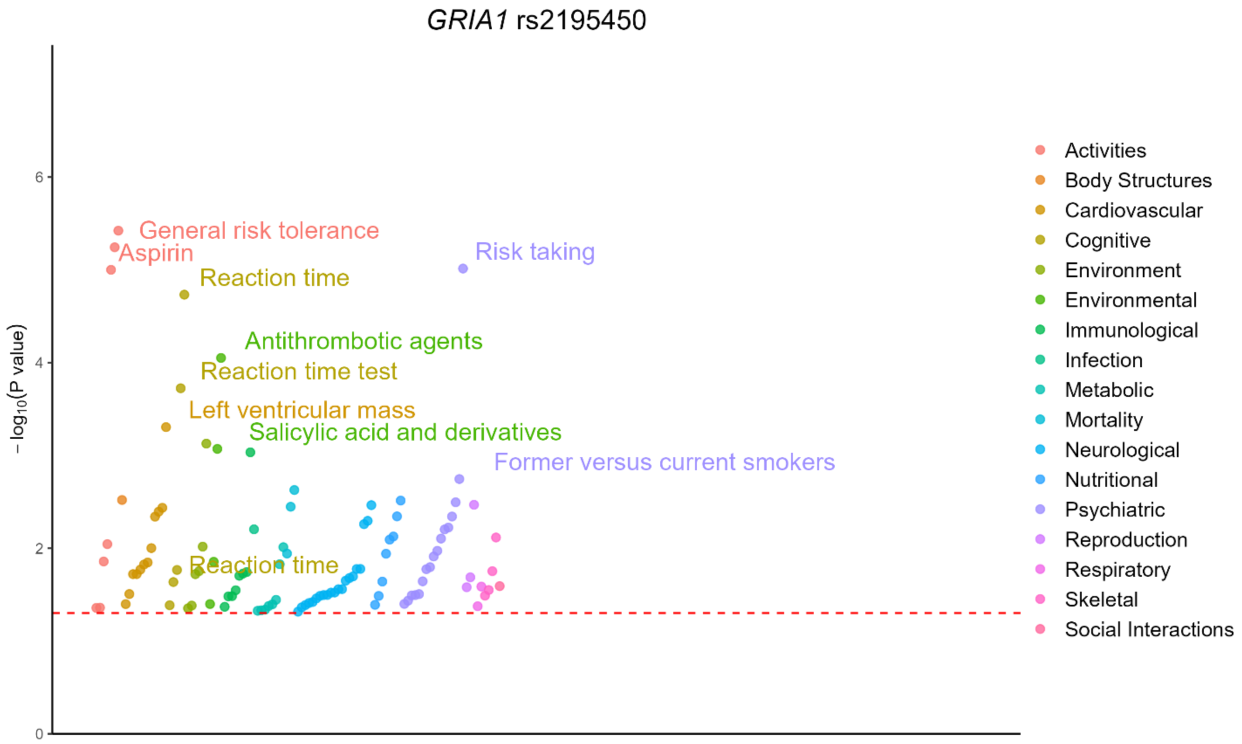

The results of the genome-wide association studies with phenotypic traits for the *GRIA1 rs2195450* variant

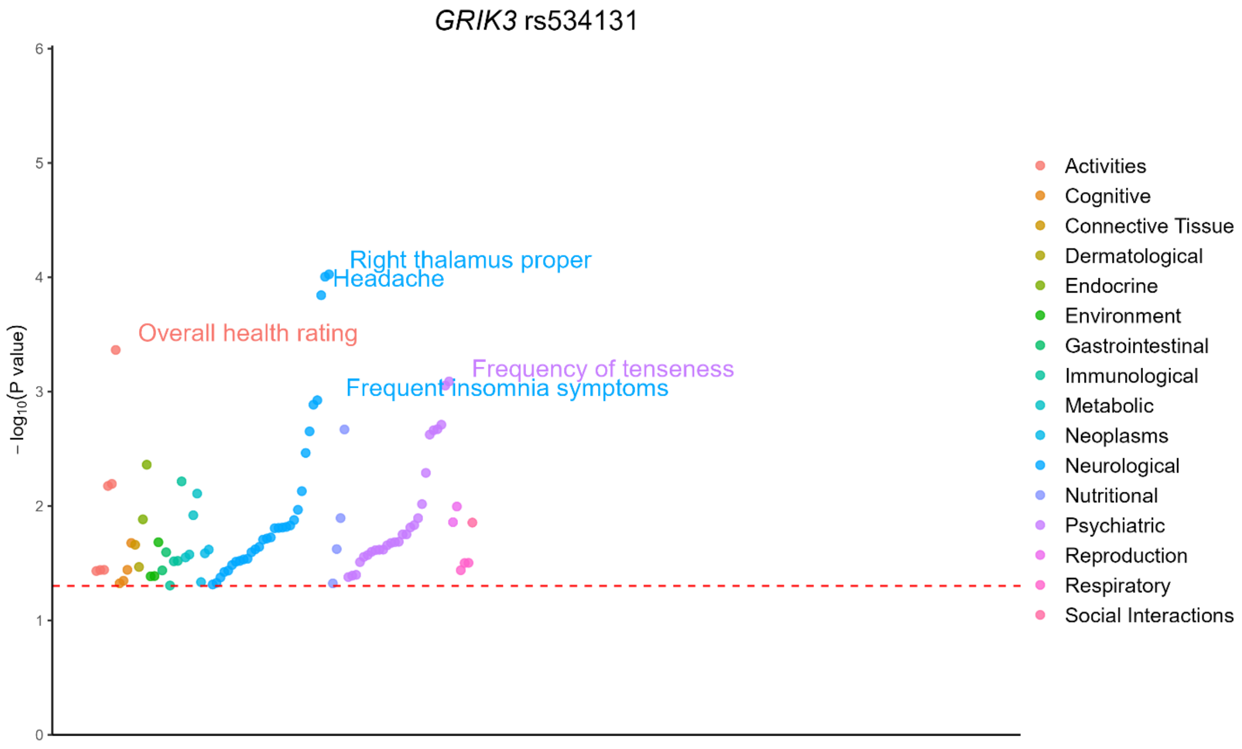

The results of the genome-wide association studies with phenotypic traits for the *GRIK3 rs534131* variant

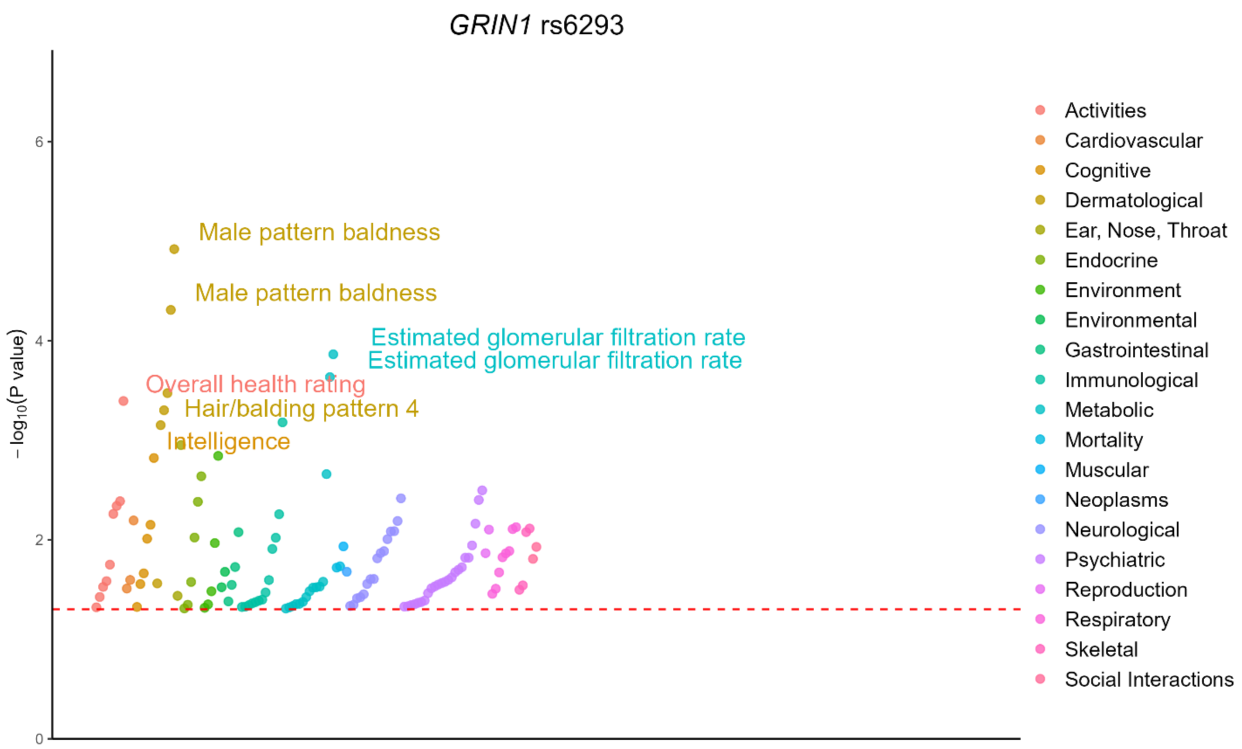

The results of the genome-wide association studies with phenotypic traits for *GRIN1 rs6293* variant

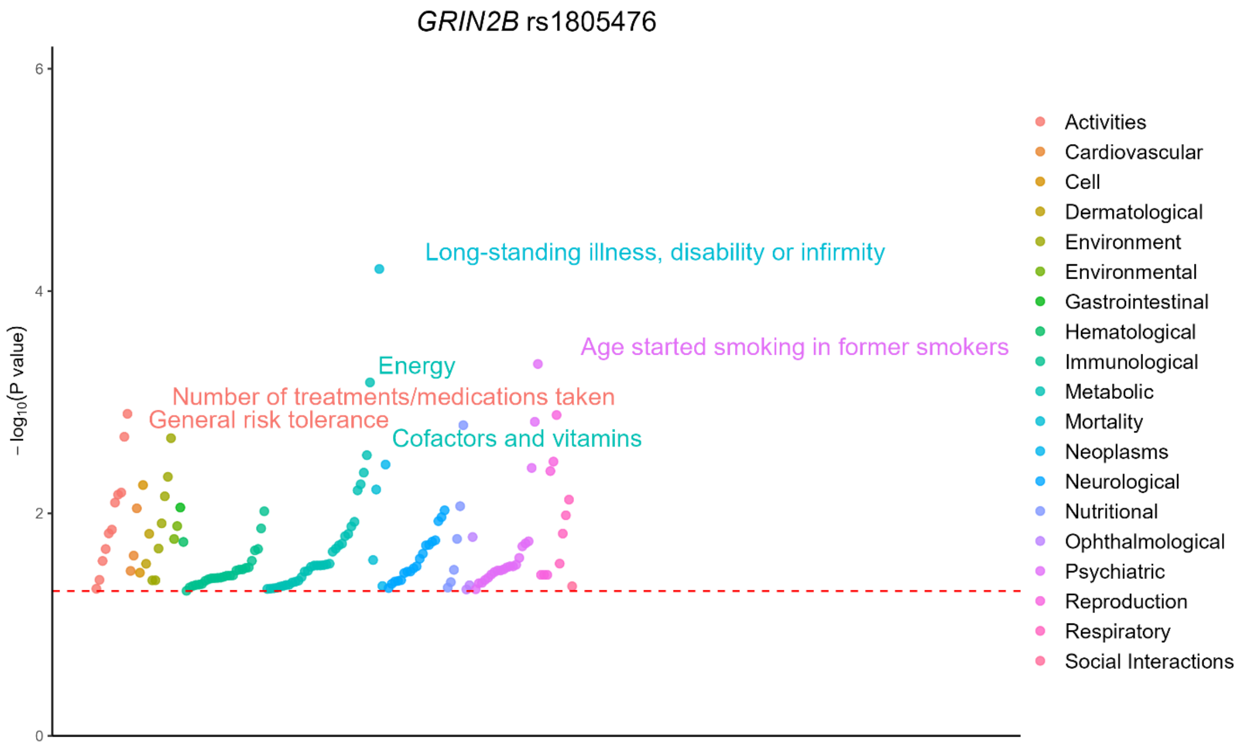

The results of the genome-wide association studies with phenotypic traits for the *GRIN2B rs1805476* variant

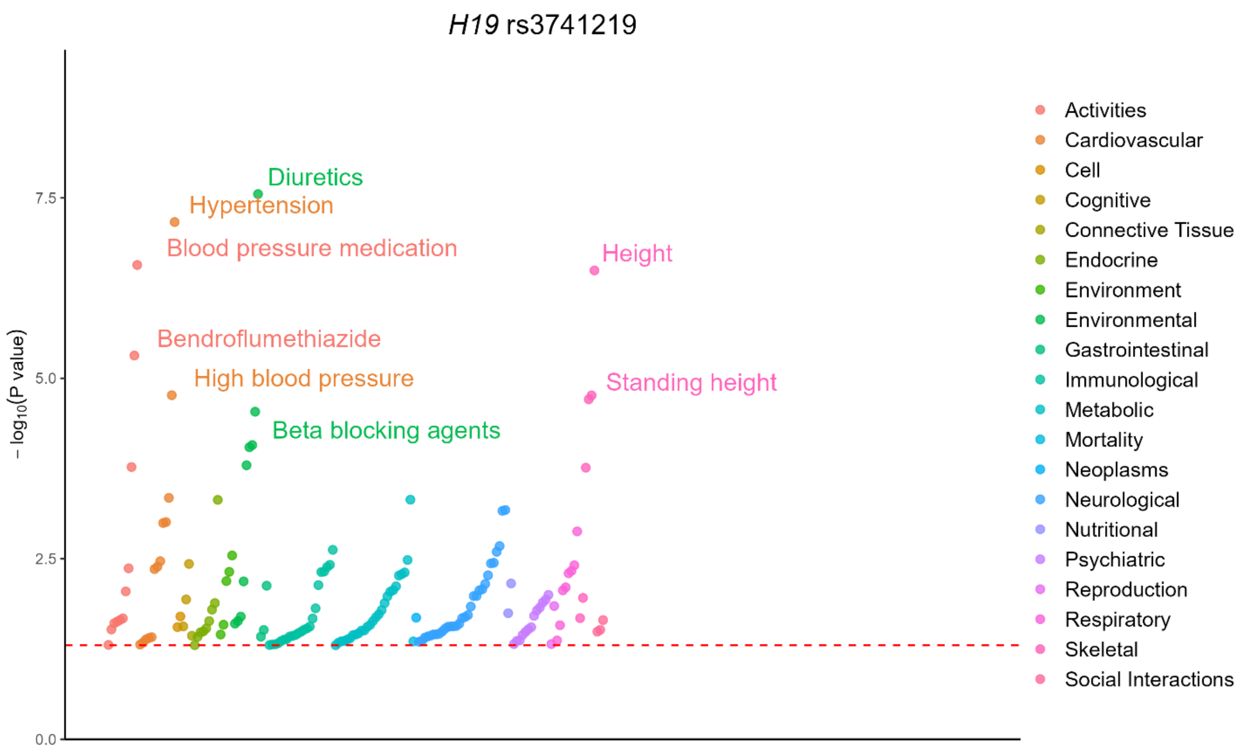

The results of the genome-wide association studies with phenotypic traits for the *H19 rs3741219* variant

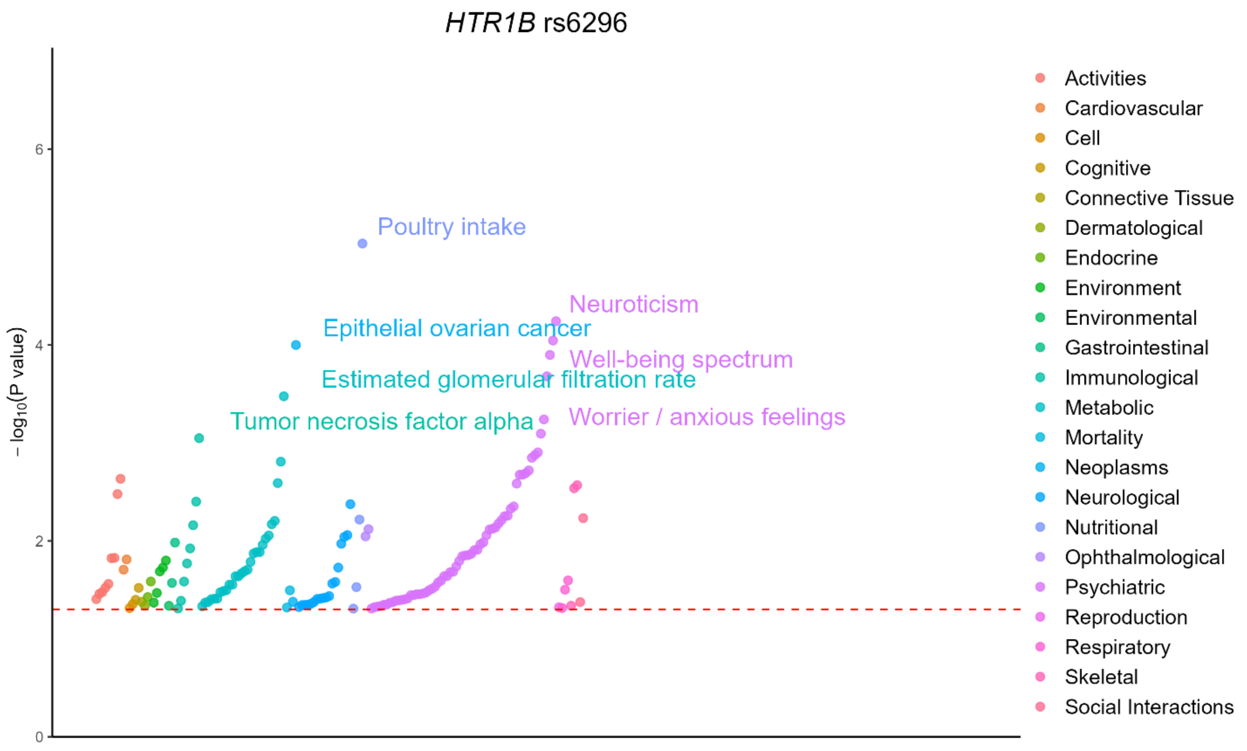

The results of the genome-wide association studies with phenotypic traits for the *HTR1B rs6296* variant

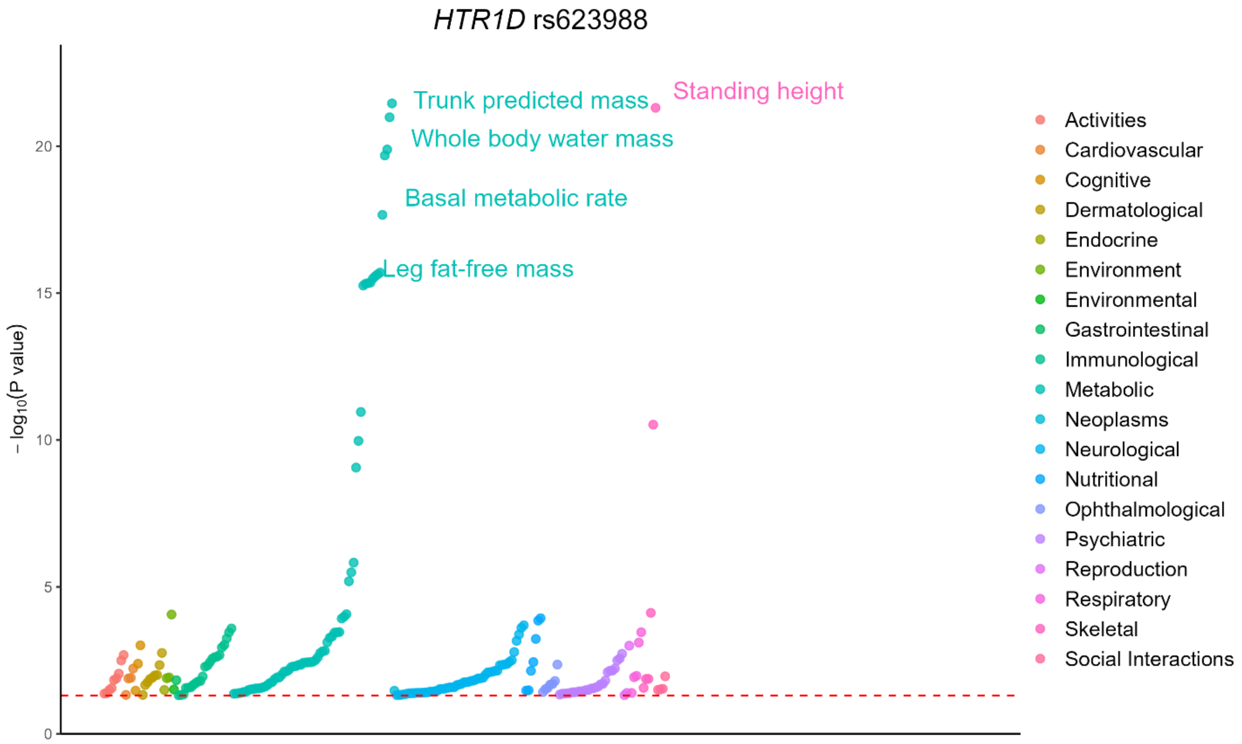

The results of the genome-wide association studies with phenotypic traits for the *HTR1D rs623988* variant

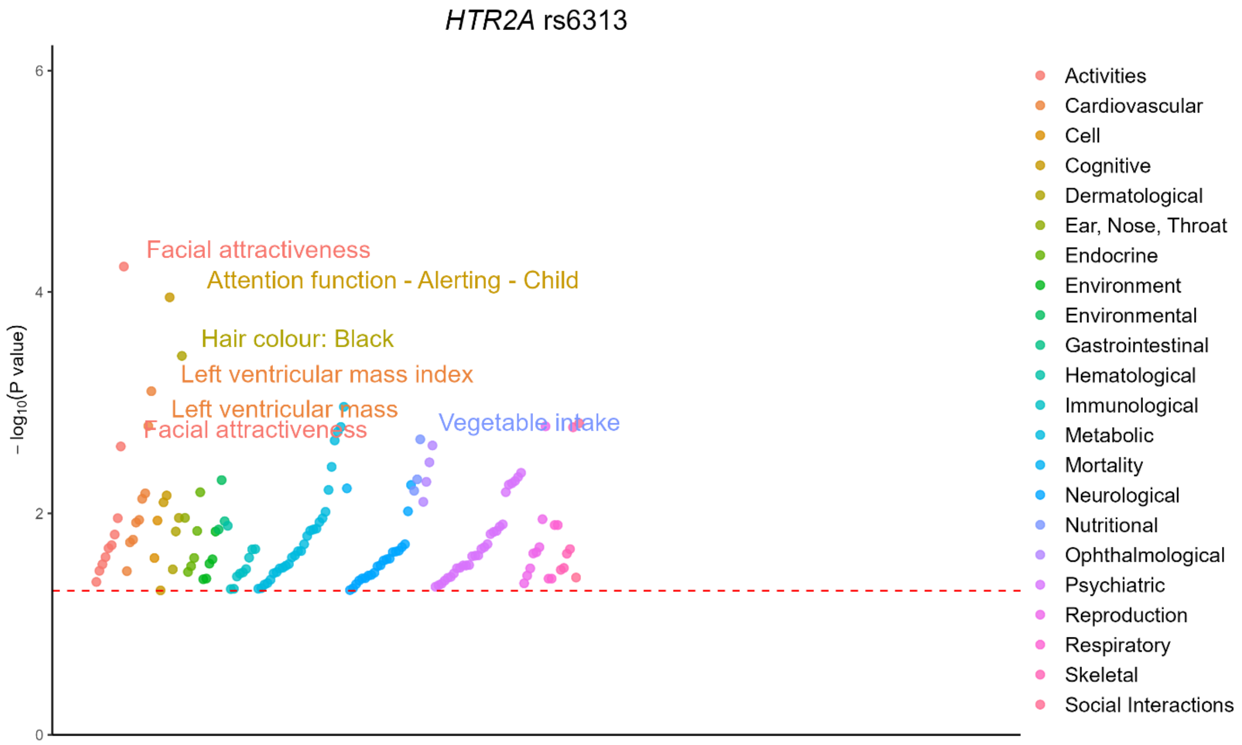

The results of the genome-wide association studies with phenotypic traits for the *HTR2A rs6313* variant

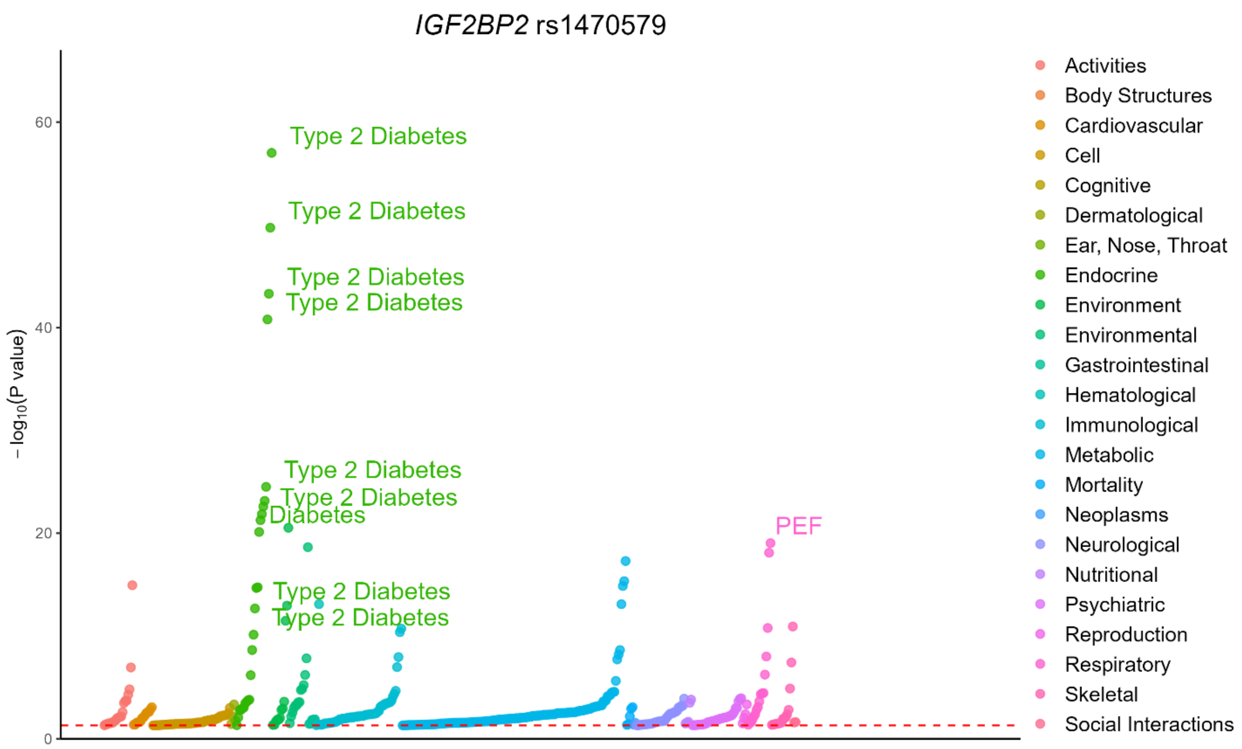

The results of the genome-wide association studies with phenotypic traits for the *IGF2BP2 rs1470579* variant

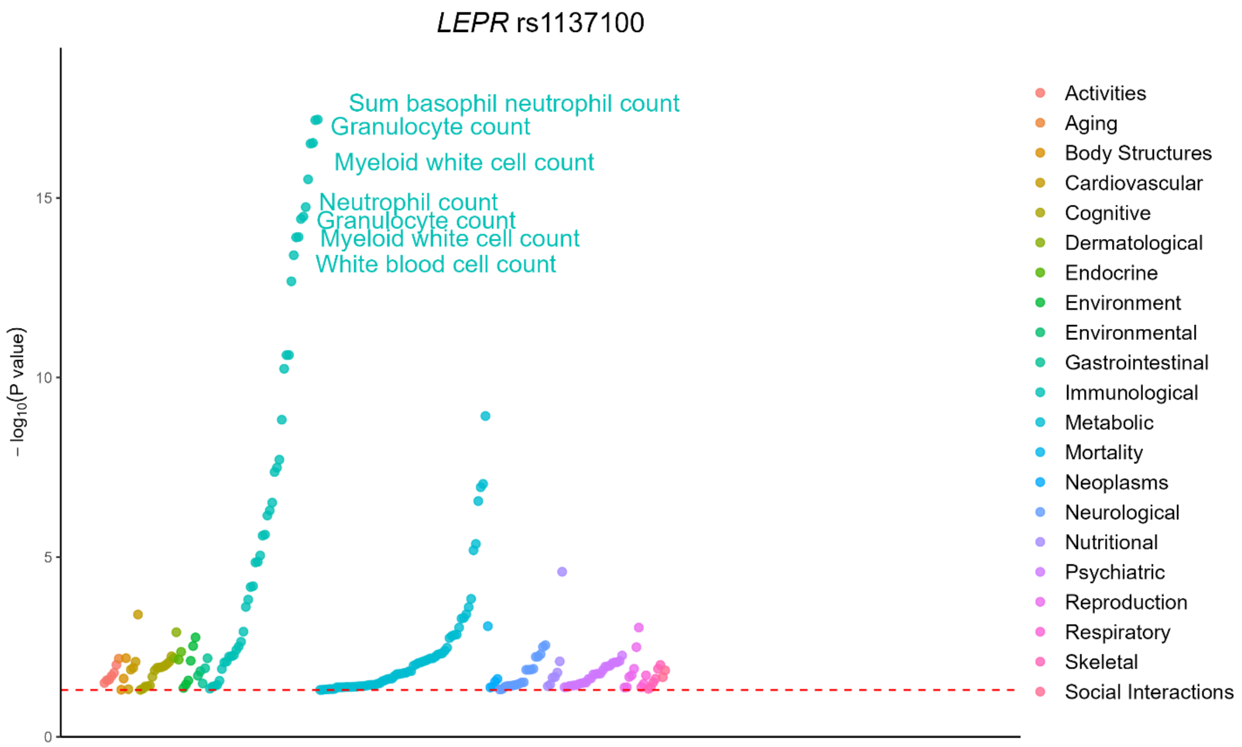

The results of the genome-wide association studies with phenotypic traits for the *LEPR rs1137100* variant

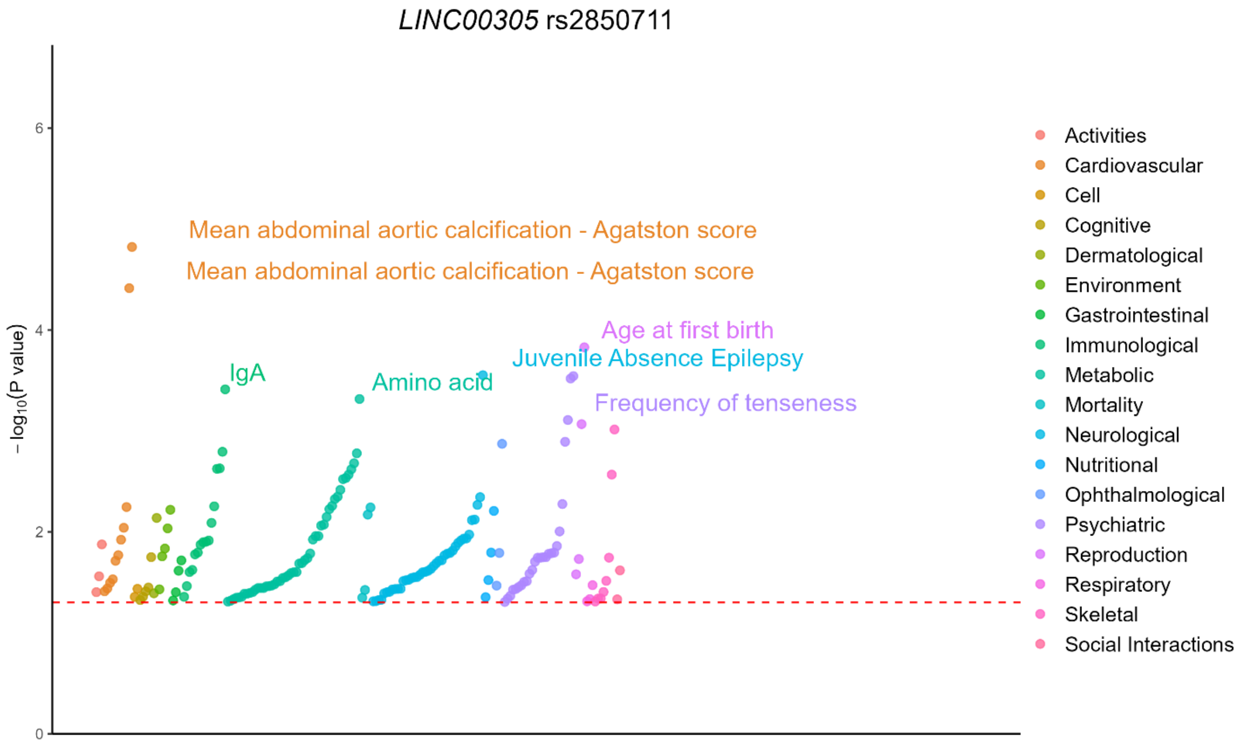

The results of the genome-wide association studies with phenotypic traits for the *LINC00305 rs2850711* variant

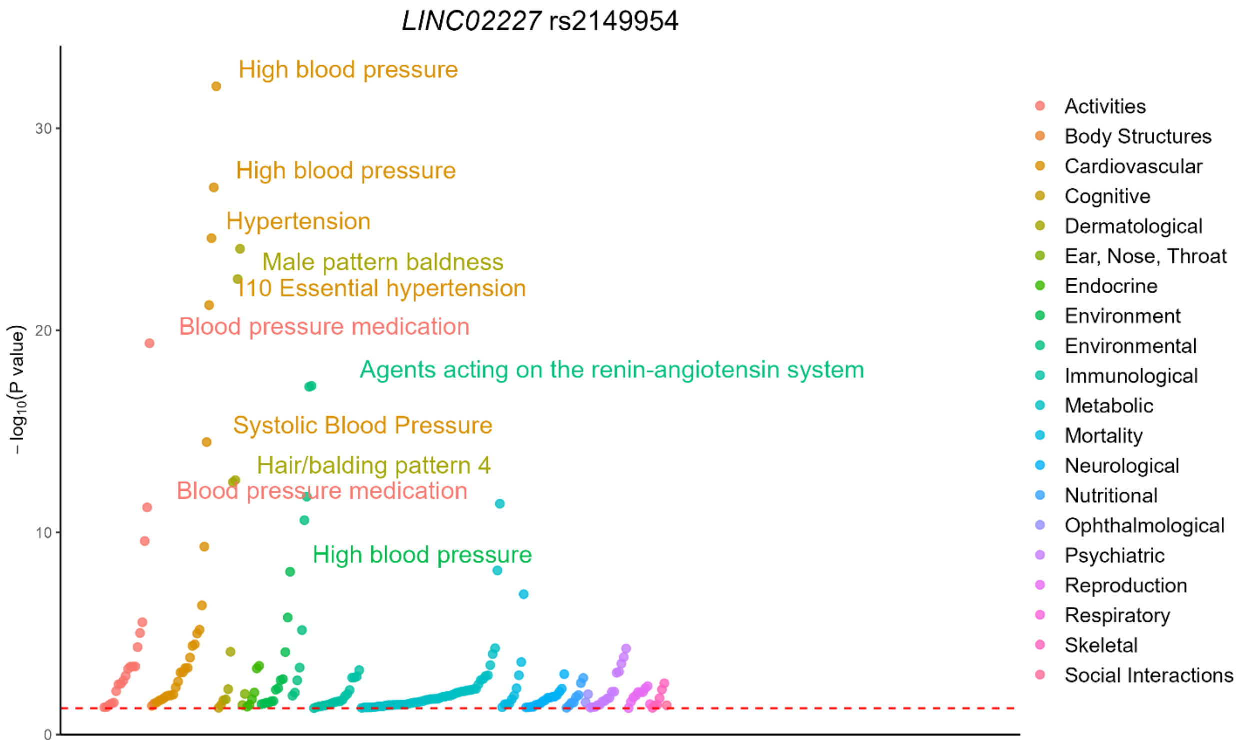

The results of the genome-wide association studies with phenotypic traits for *LINC02227 rs2149954* variant

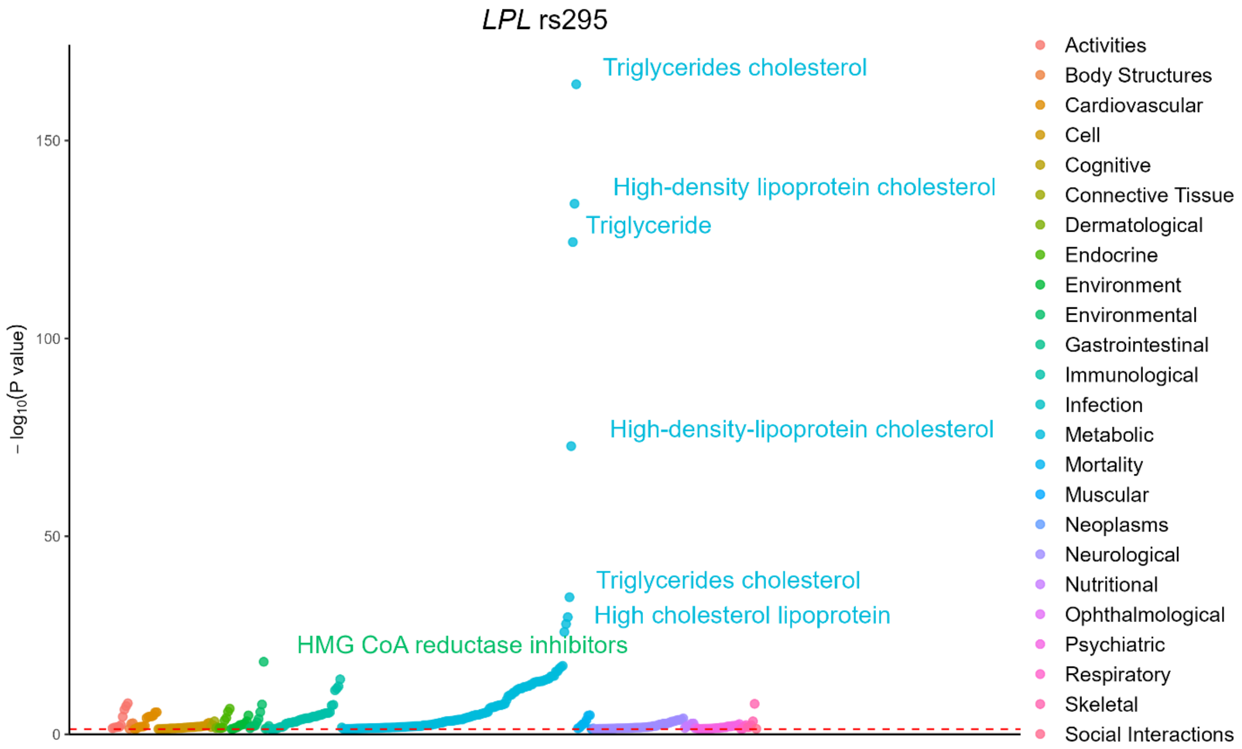

The results of the genome-wide association studies with phenotypic traits for the *LPL rs295* variant

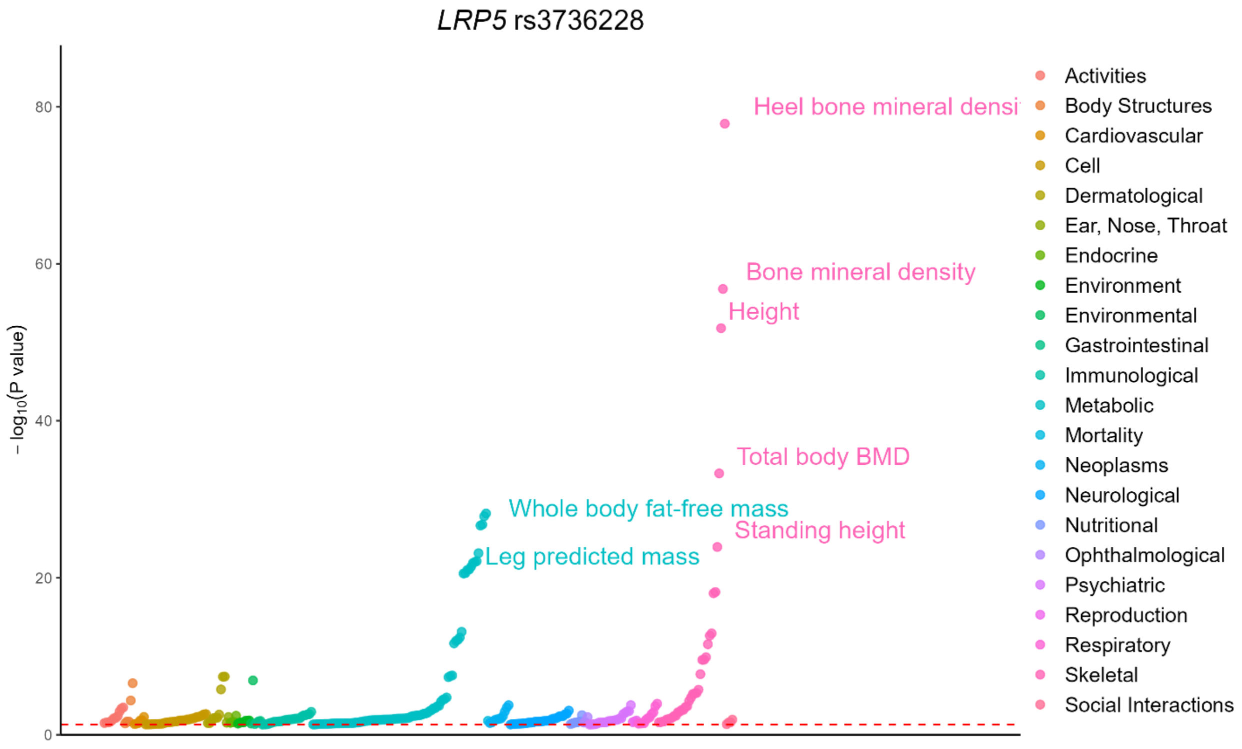

The results of the genome-wide association studies with phenotypic traits for the *LRP5 rs3736228* variant

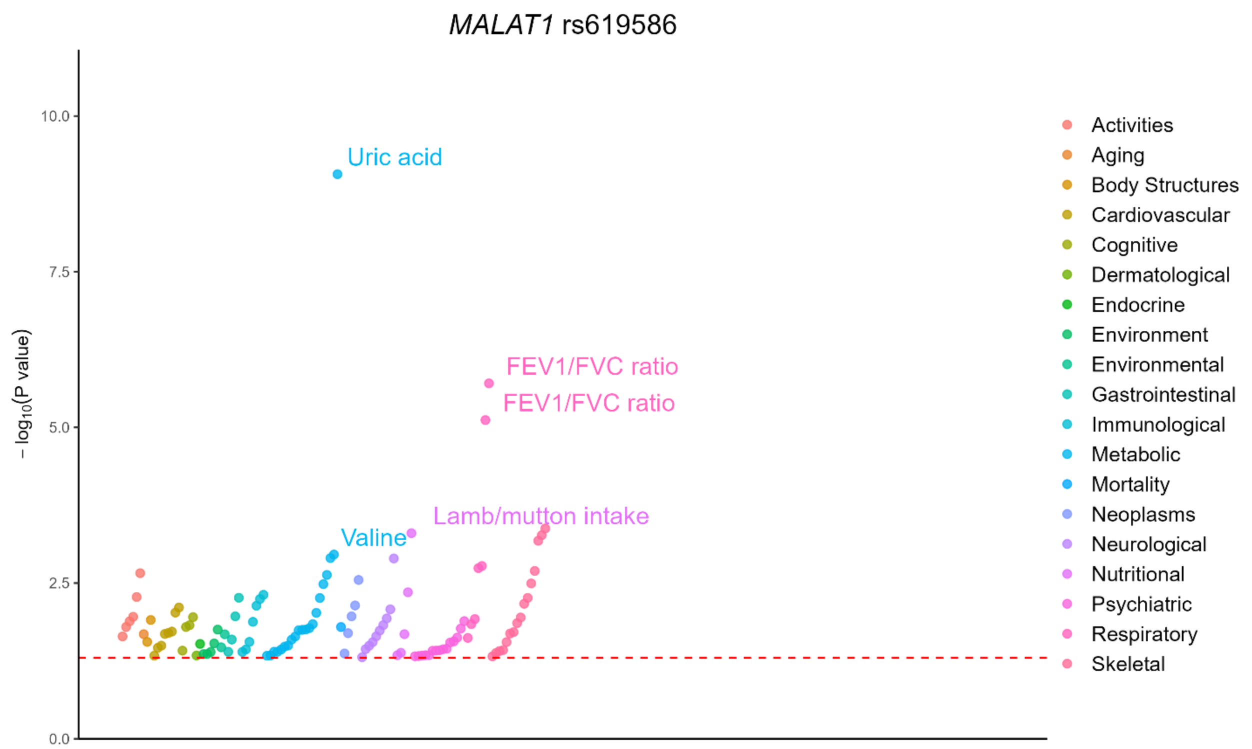

The results of the genome-wide association studies with phenotypic traits for the *MALAT1 rs619586* variant

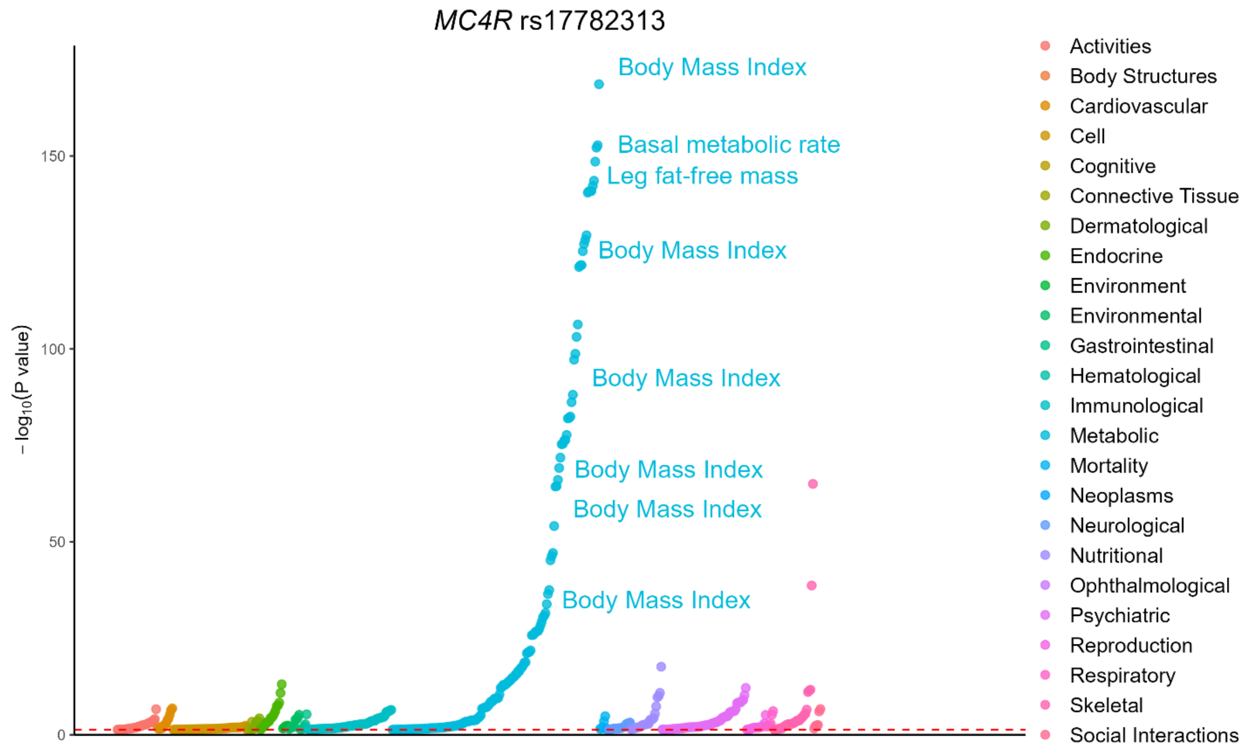

The results of the genome-wide association studies with phenotypic traits for the *MC4R rs17782313* variant

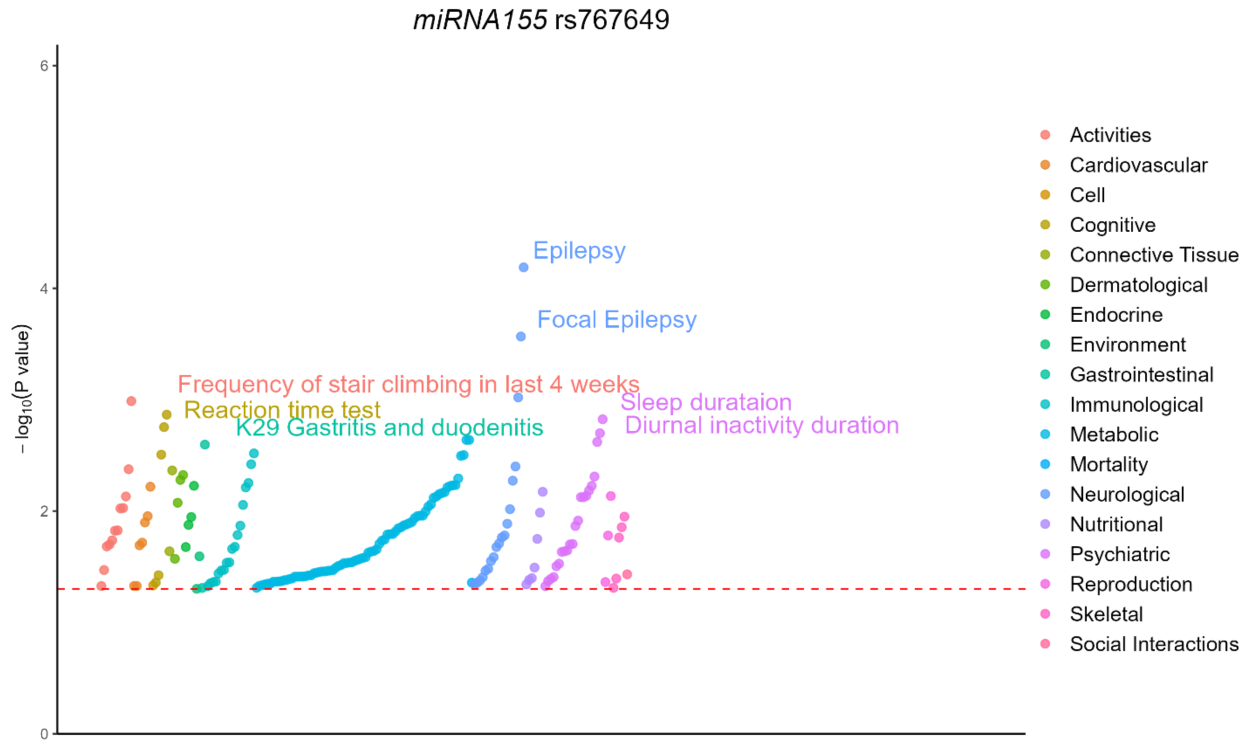

The results of the genome-wide association studies with phenotypic traits for the *miRNA155 rs767649* variant

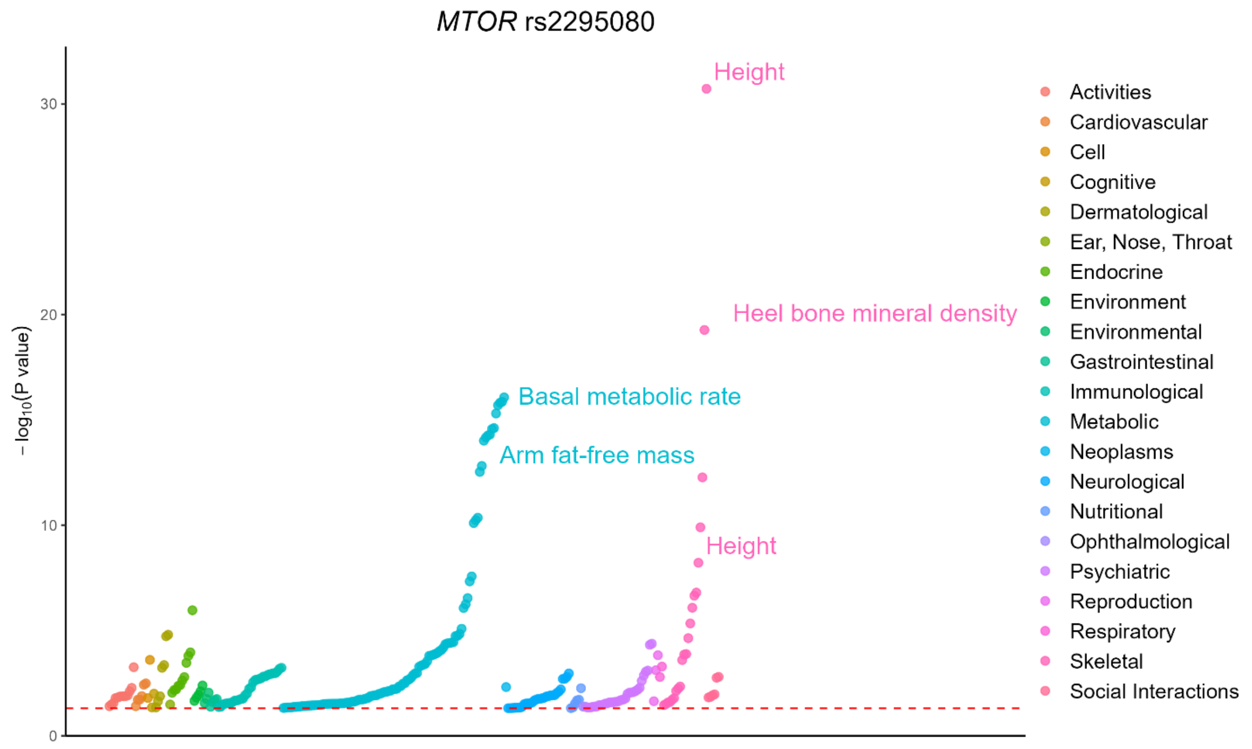

The results of the genome-wide association studies with phenotypic traits for the *MTOR rs2295080* variant

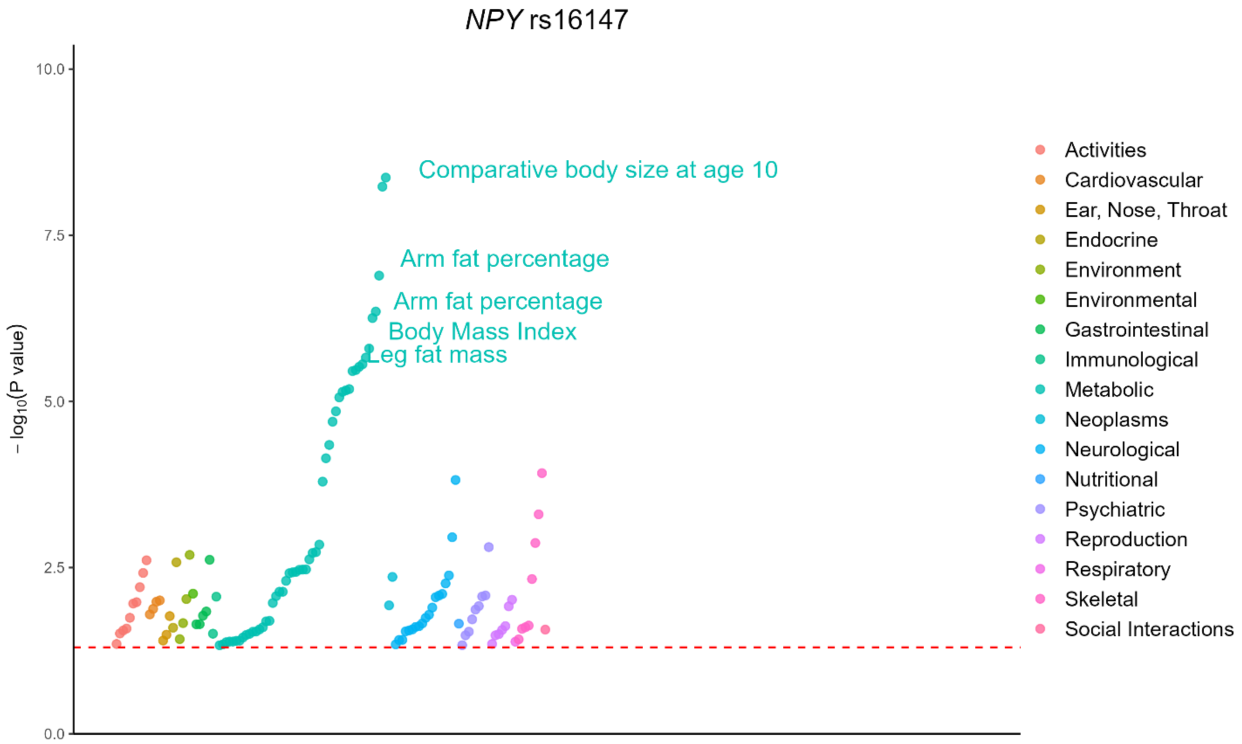

The results of the genome-wide association studies with phenotypic traits for the *NPY rs16147* variant

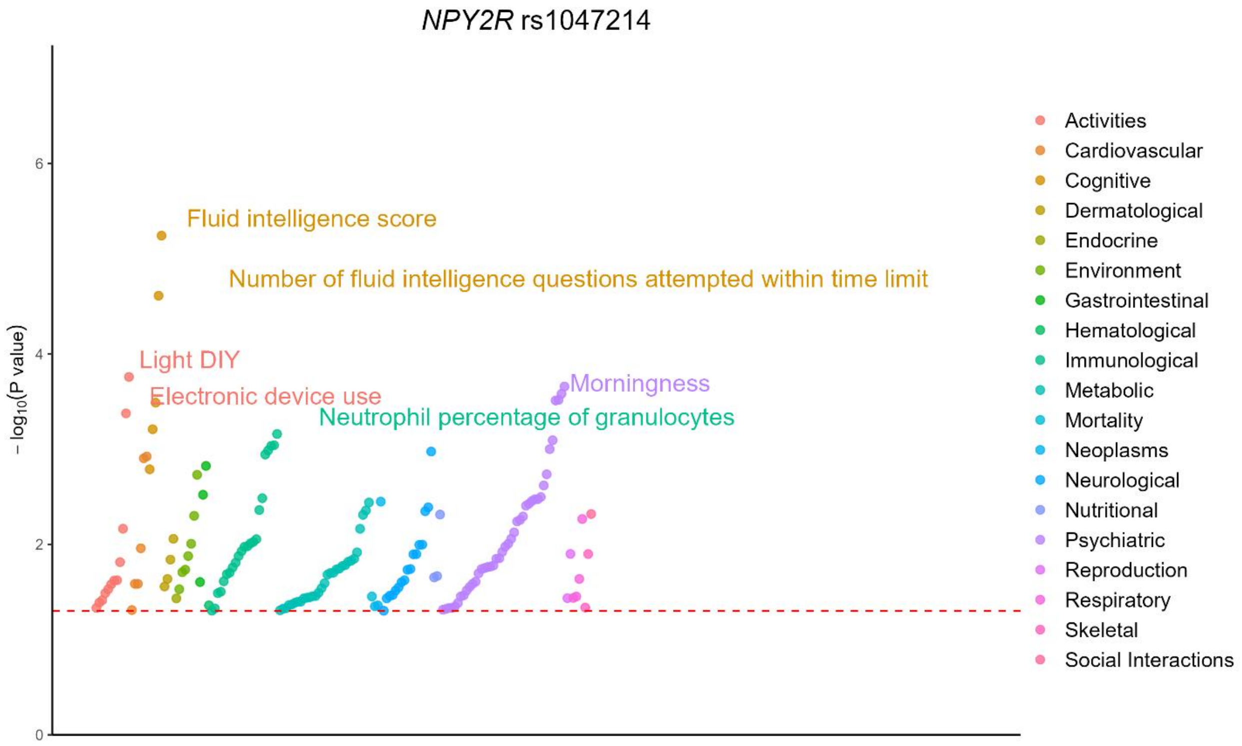

The results of the genome-wide association studies with phenotypic traits for the *NPY2R rs1047214* variant

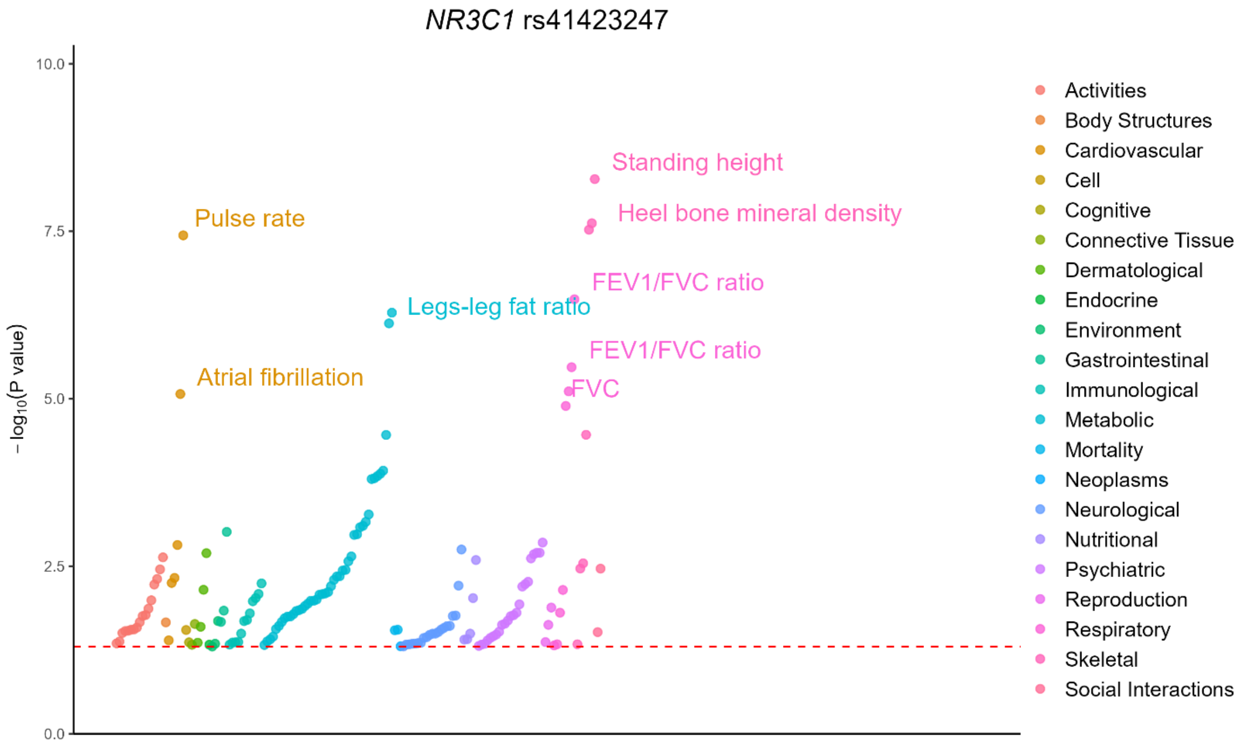

The results of the genome-wide association studies with phenotypic traits for the *NR3C1 rs41423247* variant

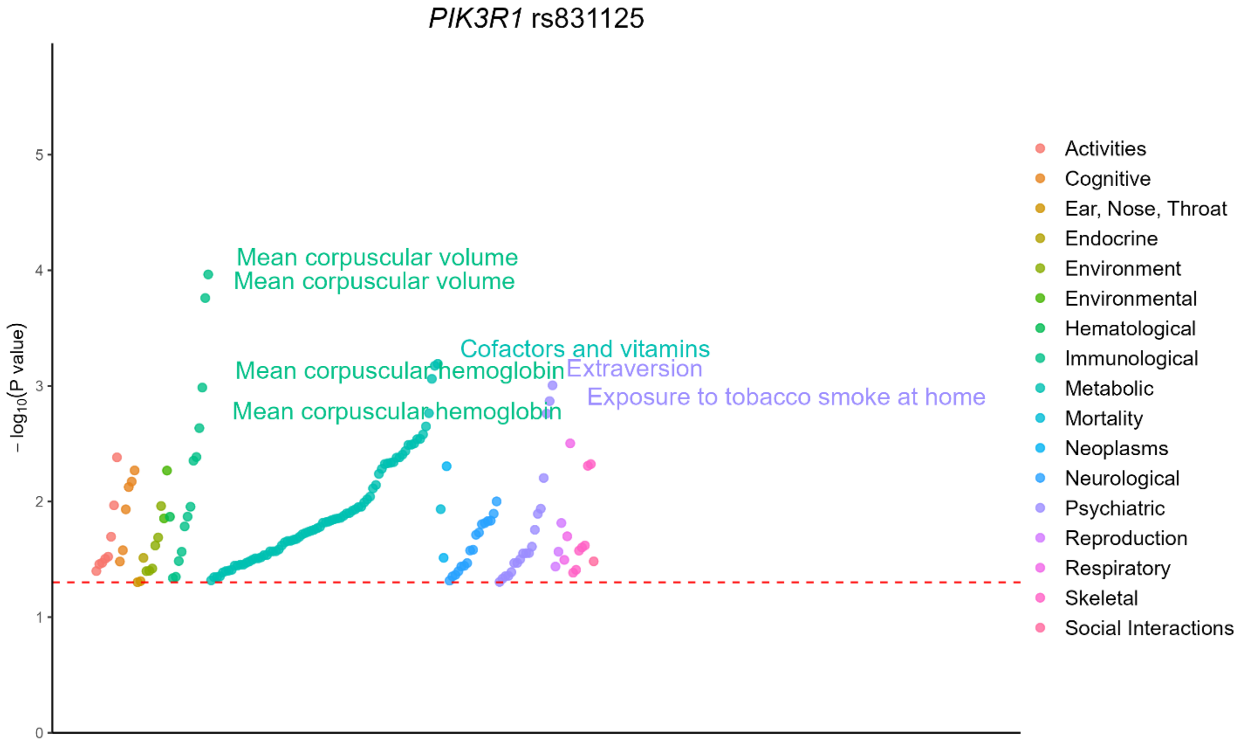

The results of the genome-wide association studies with phenotypic traits for the *PIK3R1 rs831125* variant

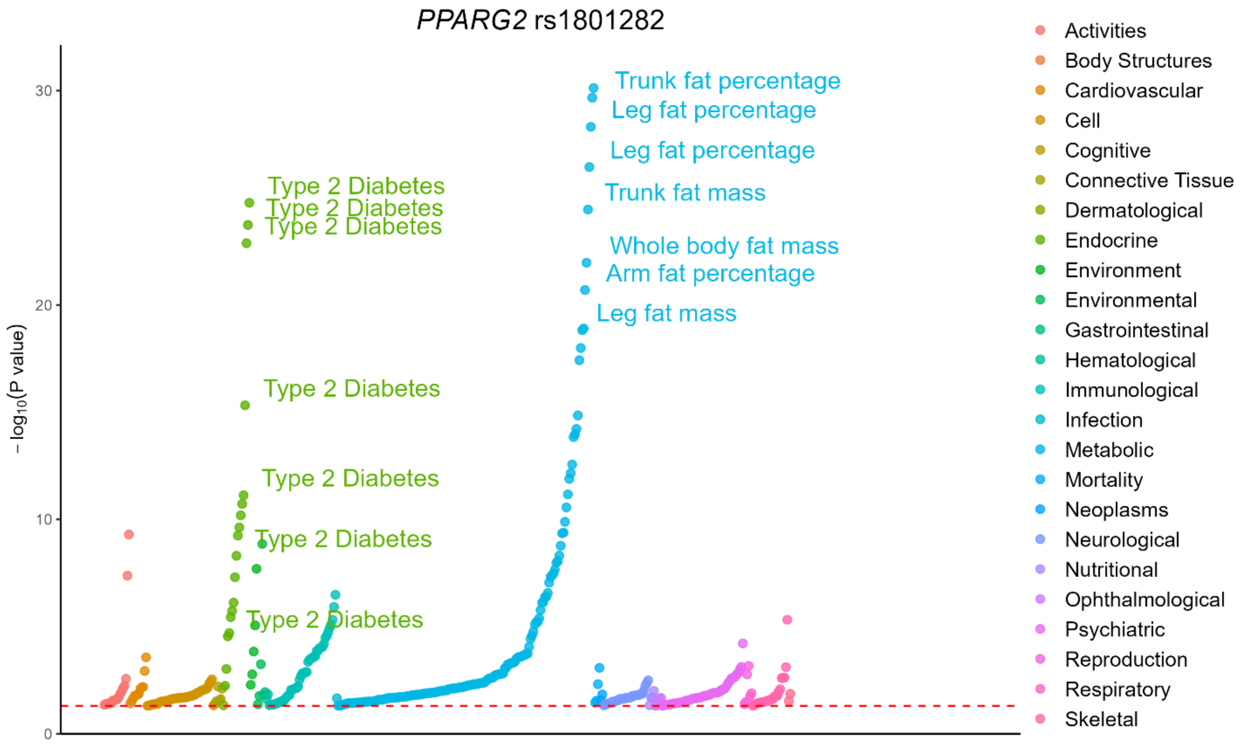

The results of the genome-wide association studies with phenotypic traits for the *PPARG2 rs1801282* variant

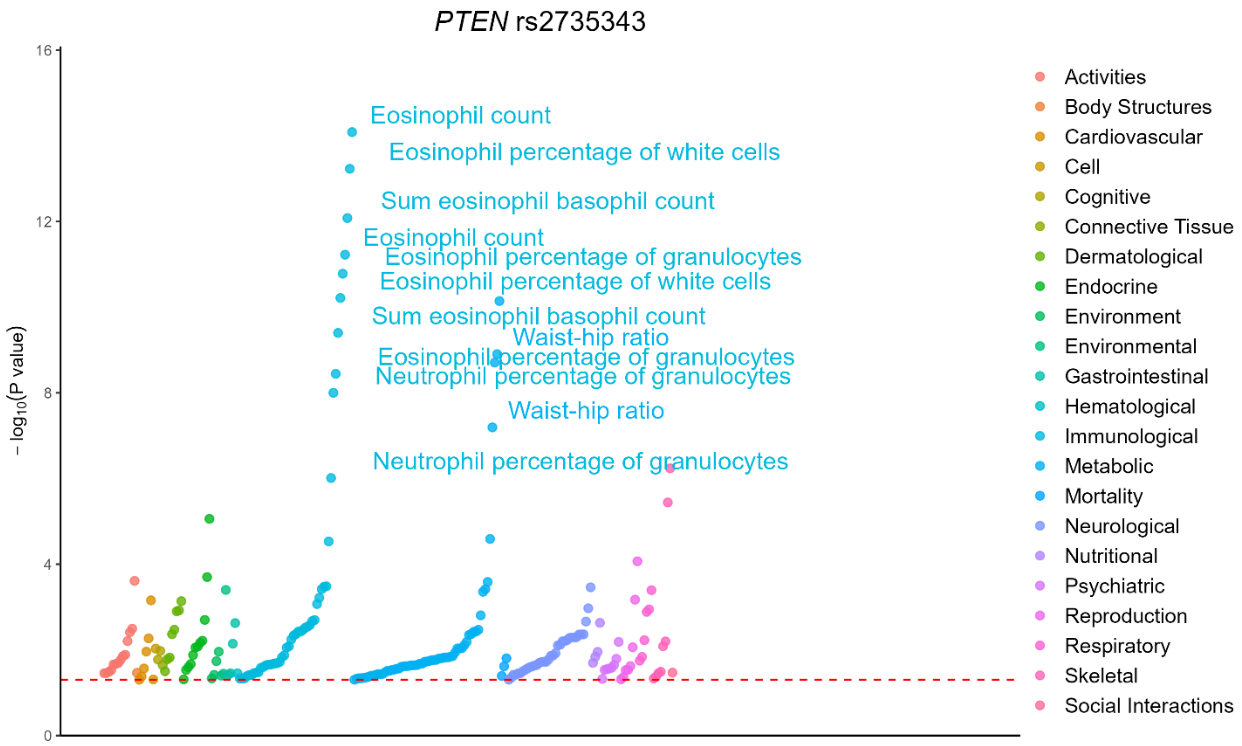

The results of the genome-wide association studies with phenotypic traits for the *PTEN rs2735343* variant

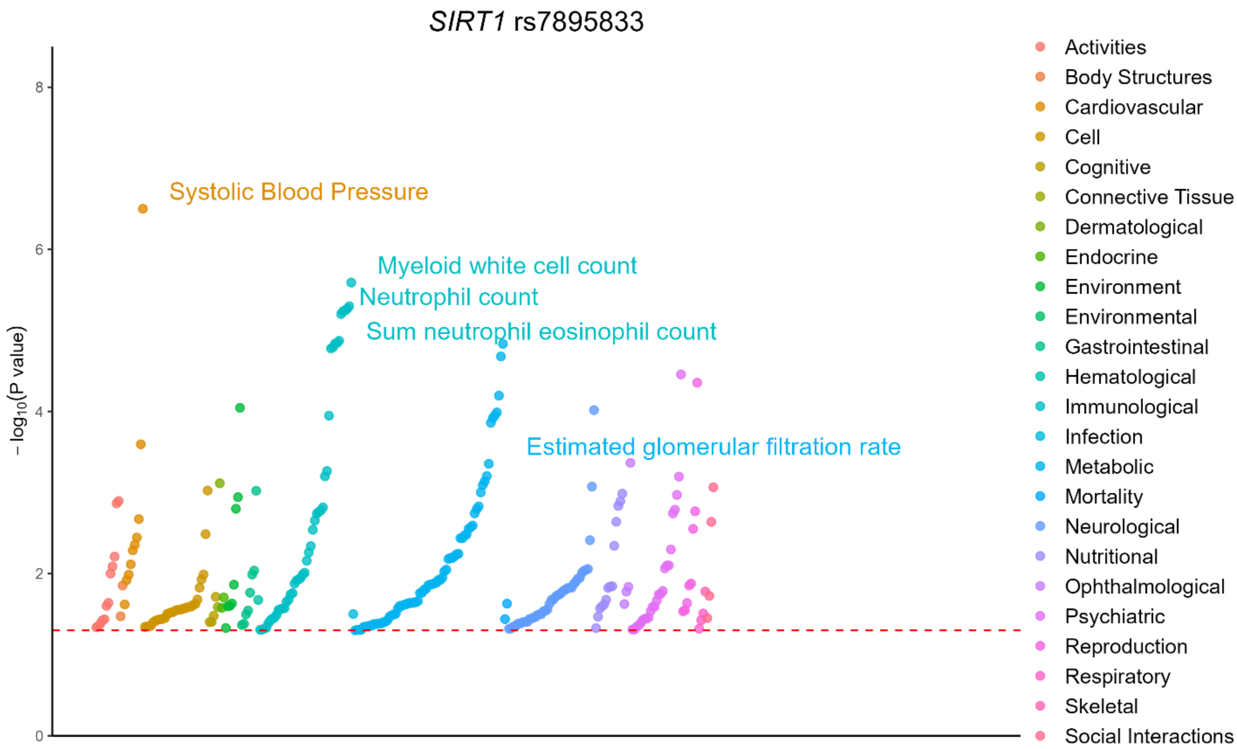

The results of the genome-wide association studies with phenotypic traits for the *SIRT1 rs7895833* variant

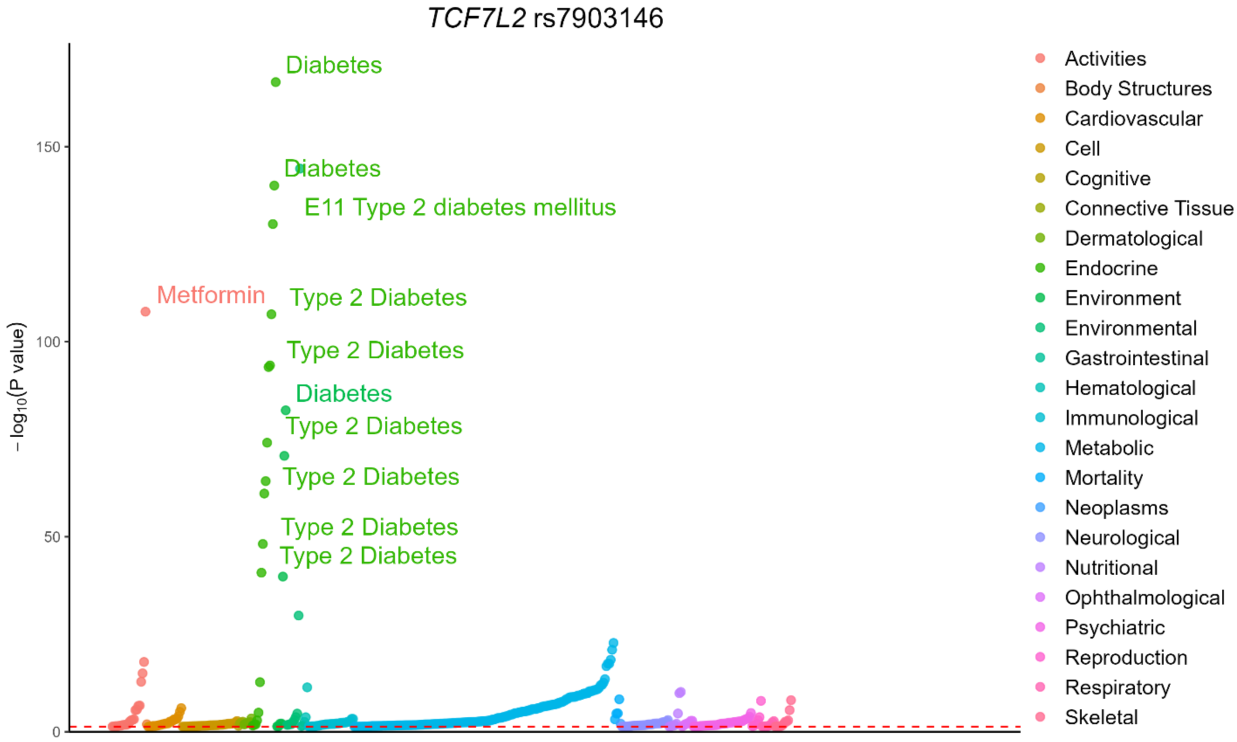

The results of the genome-wide association studies with phenotypic traits for the *TCF7L2 rs7903146* variant

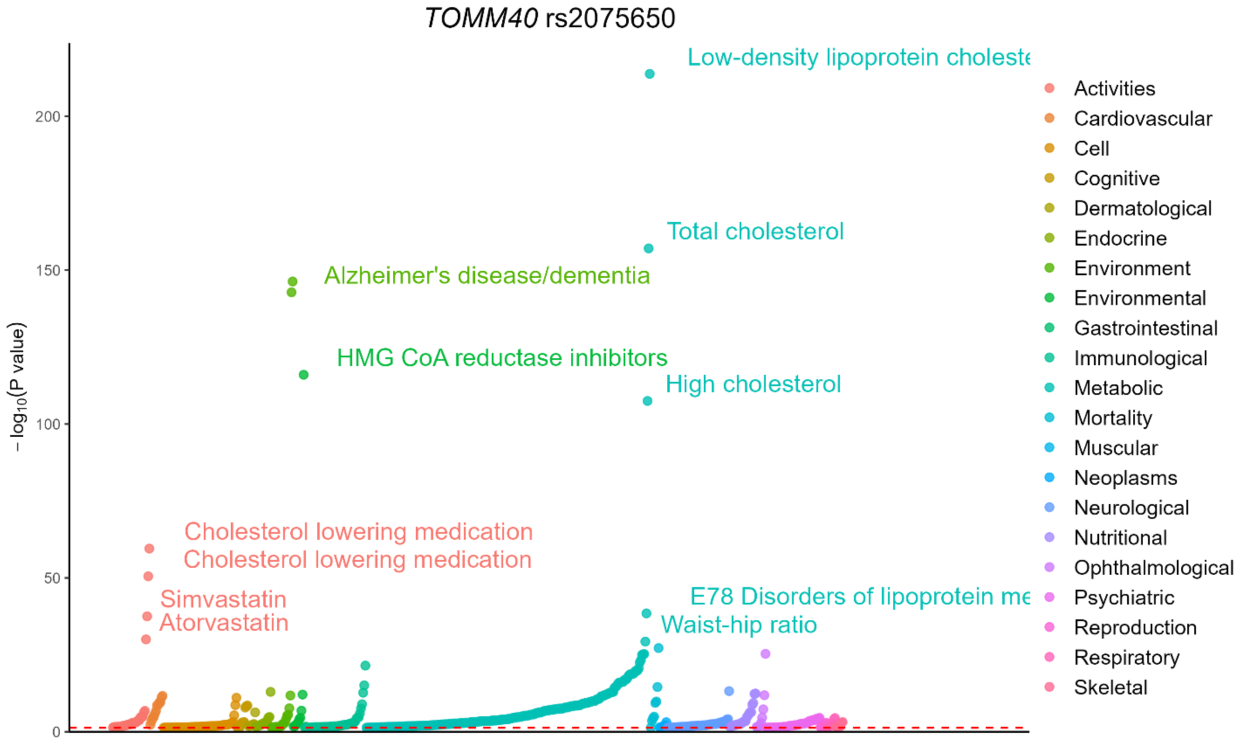

The results of the genome-wide association studies with phenotypic traits for *TOMM40 rs2075650* variant
